# Supplementary material for: Inequalities in birth before arrival at hospital in South West England: a multimethods study of neonatal hypothermia and emergency medical services call-handler advice
Source: BMJ Open. 2024 Apr 28;14(4):e081106. doi: 10.1136/bmjopen-2023-081106 (PMC11057285; doi:10.1136/bmjopen-2023-081106)
Supplement: Supplementary data [file bmjopen-2023-081106supp001.pdf]

Supplemental File 1

Definitions of characteristics of mothers and babies

- Prematurity: recorded gestation at birth (weeks and days. Pre-term (<37 weeks); Term (37+ weeks).
- Previous live birth: self-reported by the mother and identified by the midwife.
- Disability reported at booking: any disability self-reported by the mother at the booking appointment.
- Safeguarding concerns reported at booking: any safeguarding concerns self-reported by the mother or identified by the midwife at the booking appointment.
- Gestation at booking: gestational age (weeks and days) recorded at the booking appointment.
- Mother's ethnicity: self-reported by the mother at the booking appointment.
- Mother born in UK: self-reported by the mother at the booking appointment.
- Model of care: recorded by the midwife.
- Mother's attendance at antenatal appointments: recorded by the midwife.
- Mother's age at booking: recorded by the midwife.
