# Supplementary material for: Inequalities in birth before arrival at hospital in South West England: a multimethods study of neonatal hypothermia and emergency medical services call-handler advice
Source: BMJ Open. 2024 Apr 28;14(4):e081106. doi: 10.1136/bmjopen-2023-081106 (PMC11057285; doi:10.1136/bmjopen-2023-081106)
Supplement: Author's manuscript [file bmjopen-2023-081106.draft_revisions.pdf]

BMJ Open is committed to open peer review. As part of this commitment we make the peer review history of every article we publish publicly available.

When an article is published we post the peer reviewers' comments and the authors' responses online. We also post the versions of the paper that were used during peer review. These are the versions that the peer review comments apply to.

The versions of the paper that follow are the versions that were submitted during the peer review process. They are not the versions of record or the final published versions. They should not be cited or distributed as the published version of this manuscript.

BMJ Open is an open access journal and the full, final, typeset and author-corrected version of record of the manuscript is available on our site with no access controls, subscription charges or pay-per-view fees (http://bmjopen.bmj.com).

If you have any questions on BMJ Open's open peer review process please email info.bmjopen@bmj.com

BMJ Open

Inequalities in birth before arrival at hospital in South West England: a multi-methods study of neonatal hypothermia and Emergency Medical Services call-handler advice

Journal:	*BMJ Open*
Manuscript ID	bmjopen-2023-081106
Article Type:	Original research
Date Submitted by the Author:	18-Oct-2023
Complete List of Authors:	Goodwin, Laura; University of the West of England, School of Health and Social Wellbeing<br>McClelland, Graham; Northumbria University<br>Kirby, Kim; South Western Ambulance Service NHS Foundation Trust, Research, Audit and Quality Improvement Department; University of the West of England, School of Health and Social Wellbeing<br>Beach, Emily; University of the West of England, School of Health and Social Wellbeing<br>Bedson, Adam; South Western Ambulance Service NHS Foundation Trust<br>Benger, Jonathan; University of the West of England, School of Health and Social Wellbeing<br>Deave, Toity; University of the West of England, Centre for Child and Adolescent Health<br>Osborne, Ria; South Western Ambulance Service NHS Foundation Trust, Research, Audit & Improvement<br>McAdam, Helen; University of Sunderland, Health Sciences and Wellbeing<br>McKeon-Carter, Roisin; University Hospitals Plymouth NHS Trust<br>Miller, Nick; University of the West of England, School of Health and Social Wellbeing<br>Taylor, Hazel; University Hospitals Bristol NHS Foundation Trust, Research Design Service<br>Voss, Sarah; University of the West of England, School of Health and Social Wellbeing
Keywords:	ACCIDENT & EMERGENCY MEDICINE, Health Equity, NEONATOLOGY, Maternal medicine < OBSTETRICS, Paediatric A&E and ambulatory care < PAEDIATRICS

SCHOLARONE™
Manuscripts

I, the Submitting Author has the right to grant and does grant on behalf of all authors of the Work (as defined in the below author licence), an exclusive licence and/or a non-exclusive licence for contributions from authors who are: i) UK Crown employees; ii) where BMJ has agreed a CC-BY licence shall apply, and/or iii) in accordance with the terms applicable for US Federal Government officers or employees acting as part of their official duties; on a worldwide, perpetual, irrevocable, royalty-free basis to BMJ Publishing Group Ltd ("BMJ") its licensees and where the relevant Journal is co-owned by BMJ to the co-owners of the Journal, to publish the Work in this journal and any other BMJ products and to exploit all rights, as set out in our licence.

The Submitting Author accepts and understands that any supply made under these terms is made by BMJ to the Submitting Author unless you are acting as an employee on behalf of your employer or a postgraduate student of an affiliated institution which is paying any applicable article publishing charge ("APC") for Open Access articles. Where the Submitting Author wishes to make the Work available on an Open Access basis (and intends to pay the relevant APC), the terms of reuse of such Open Access shall be governed by a Creative Commons licence – details of these licences and which Creative Commons licence will apply to this Work are set out in our licence referred to above.

Other than as permitted in any relevant BMJ Author's Self Archiving Policies, I confirm this Work has not been accepted for publication elsewhere, is not being considered for publication elsewhere and does not duplicate material already published. I confirm all authors consent to publication of this Work and authorise the granting of this licence.

For peer review only

Inequalities in birth before arrival at hospital in South West England: a multi-methods study of neonatal hypothermia and Emergency Medical Services call-handler advice

Authors:
Goodwin, L.[1], McClelland, G.[2], Kirby, K.[1,3] Beach, E.[1], Bedson, A.[3], Benger, J.[1], Deave, T.[1], Osborne, R.[3], McAdam, H.[4], McKeon-Carter, R.[5], Miller, N.[1], Taylor, H.[6], & Voss, S.[1]

Institutions:
[1] University of the West of England, Bristol
[2] Northumbria University
[3] South Western Ambulance Service NHS Foundation Trust
[4] University of Sunderland
[5] University Hospitals Plymouth NHS Trust
[6] University Hospitals Bristol and Weston NHS Foundation Trust

Corresponding author:
Laura Goodwin – laura.goodwin@uwe.ac.uk

Keywords: Infant, Newborn, Ambulances, Hypothermia, Temperature, Emergency Service, Hospital

List of abbreviations:
AHSN – Academic Health Science Network
BBA – Birth Before Arrival at hospital
PPI – Patient and Public Involvement
SWASFT – South Western Ambulance Service NHS Foundation Trust
UK – United Kingdom

Word count: 4266

For peer review only - http://bmjopen.bmj.com/site/about/guidelines.xhtml

ABSTRACT

Objectives: To examine inequalities in birth before arrival (BBA) at hospital in South West England and understand which groups are most likely to experience BBA, and how this relates to hypothermia and outcomes (Phase A). To investigate opportunities to improve temperature management advice given by Emergency Medical Services (EMS) call-handlers during emergency calls regarding BBA in the United Kingdom (Phase B).

Design: A two-phase multi-method study. Phase A analysed anonymised data from hospital neonatal records between January 2018 and January 2021. Phase B analysed anonymised EMS call transcripts, followed by focus groups with National Health Service (NHS) staff and patients.

Setting: Six Hospital Trusts in South West England, and two EMS providers (ambulance services) in South West and North East England.

Participants: Eighteen multidisciplinary NHS staff and 22 members of the public who had experienced BBA in the UK.

Results: 35% (64/184) of babies conveyed to hospital were hypothermic on arrival. When compared with national data on all births in the South West, we found higher percentages of women with documented safeguarding concerns at booking, previous live births, and 'late bookers' (booking their pregnancy >13 weeks gestation). These women may therefore be more likely to experience BBA. Pre-term babies, babies to first-time mothers, and babies born to mothers with disability or safeguarding concerns at booking were more likely to be hypothermic following BBA. Five main themes emerged from qualitative data on call-handler advice: 1) the importance placed on neonatal temperature; 2) advice on where the baby should be placed following birth; 3) advice on how to keep the baby warm; 4) the timing of temperature management advice; 5) the clarity and priority of instructions.

Conclusions: Findings identified factors associated with BBA and neonatal hypothermia following BBA. Findings also identified areas for improvement in EMS call-handler advice regarding neonatal temperature management.

STRENGTHS AND LIMITATIONS OF THIS STUDY

- To the best of our knowledge, this is the first study to investigate the temperature of babies arriving at hospital following a prehospital birth in South West England
- This is the first study to investigate neonatal temperature management advice provided by emergency service call-handling triage systems
- The service evaluation of hospital neonatal records had a small sample size and was confined to one geographical area which limits the representativeness of our findings.

FUNDING STATEMENT

The study was funded by the South West Academic Health Science Network as part of its Perinatal Health Equity programme.

COMPETING INTERESTS

None declared

For peer review only

BACKGROUND

In the UK, around 3,700 births a year are prehospital births, or Births Before Arrival at hospital (BBA). Paramedics are often present at these births, which usually occur without the attendance of a qualified midwife or obstetrician. BBA is associated with unfavourable perinatal outcomes and increased mortality.[1–3] An important risk factor for mortality following BBA is hypothermia,[3–5] which is defined by the World Health Organization (WHO) as a temperature below 36.5°C.[6] The mortality risk from hypothermia is especially prominent in premature babies; in low birth weight infants, mortality increases by 28% per 1°C decrease in admission temperature below 36.5°C.[7] Paramedics are usually the first on scene at BBAs, however recent audits of Emergency Medical Service (EMS) data in the UK suggest that paramedics do not routinely take newborn temperatures (temperatures recorded in 2-10% of cases).[8,9] When temperatures have been recorded, the majority have indicated neonatal hypothermia (72%).[8,9] Currently there are no published data to show the percentage of babies who are hypothermic on arrival to hospital following a BBA, and the resulting outcomes for these babies.

In order to prevent neonatal hypothermia and poor outcomes following BBA it is important to understand which women are most likely to experience BBA, which babies are most likely to be cold on arrival, and how this interacts with outcomes such as mortality, length of hospital stay, and the need for special care and interventions. Literature around inequalities in maternal and perinatal outcomes suggests an increased risk of poor outcomes for women who do not engage or have little engagement with maternity services.[10,11] This includes concealed or teenage pregnancies, as well as women from migrant or minority ethnic backgrounds.[12,13]

In the prehospital setting, babies can become hypothermic within minutes[14] and are sometimes born before EMS arrival. In these instances, the newborn is more likely to be hypothermic on arrival at hospital, potentially due to ineffective warming by non-professionals.[15] Therefore, the earlier that effective warming measures can be initiated (for example at the point of calls to EMS), the better the chances of improved outcomes following BBA. A recent Healthcare Safety Investigation Branch (HSIB) report[16] highlighted the fact that UK investigations into poor patient outcomes following BBA do not routinely consider the clinical impact of advice given by EMS call-handlers, and recommended work with relevant stakeholders to develop guidance for maternity emergencies during these calls. It is therefore necessary to investigate the advice given regarding neonatal temperature management during EMS calls relating to BBA, and work with relevant stakeholders to investigate potential ways to develop and improve this advice.[16]

The aims of this project were:

1) To examine inequalities in BBA in South West England and understand which groups are most likely to experience BBA, and also how this relates to hypothermia and poor outcomes (Phase A).
2) To investigate opportunities to improve the temperature management advice given by EMS call-handlers during emergency calls regarding BBA in the UK (Phase B).

For peer review only - http://bmjopen.bmj.com/site/about/guidelines.xhtml

METHODS

Setting

Hospital data were collected from six NHS Hospital Trusts in South West England. The area covered by these Trusts has one of the lowest population densities in the country, with people living across a mix of coastal communities, isolated rural areas, and urban centres.[17]

Data for EMS calls were collected from two NHS ambulance service Trusts using the two different EMS triage providers used in England: the Advanced Medical Priority Dispatch System (AMPDS) (Salt Lake City, USA) and NHS Pathways (NHS Digital, Leeds, UK). Data from calls triaged by AMPDS were collected from the South Western Ambulance Service NHS Foundation Trust (SWASFT), while data from calls triaged by NHS Pathways were collected from the North East Ambulance Service NHS Foundation Trust (NEAS). AMPDS has been in use in the United States since 1978 and is used by emergency services in 59 countries, including roughly half of ambulance service Trusts in the UK.[16] NHS Pathways is used by the other UK ambulance trusts and is overseen by a National Clinical Governance Group (NCGG), which is hosted by the Royal College of General Practitioners and is made up of representatives from UK medical royal colleges.[16]

Patient and public involvement (PPI)

A dedicated PPI group guided this work from its inception. The group consisted of four women and two men who had experience of BBA. Experiences of the team were varied and represented ethnic, cultural and geographical diversity. Over the course of the project, the PPI group reviewed and refined participant facing documents for stakeholder focus groups, contributed to data synthesis, and advised the team on the approach to recruitment and dissemination.

Study design

This multi-methods study consisted of both qualitative and quantitative data analyses, with two phases. Phase A included an analysis of hospital data and Phase B included content analysis of EMS calls, followed by focus groups with NHS staff and patients.

Phase A: Neonatal hypothermia on arrival at hospital

Anonymised extracts from routinely collected non-identifiable data (hospital neonatal records for BBA babies) were provided by participating Trusts from a three-year period (January 2018-January 2021). These were compared with National Maternity Data from the same geographical area to understand which groups are most likely to experience BBA and how this relates to hypothermia and poor outcomes.

Recruitment

Hospital Trusts were eligible to take part if they were covered by the South West Academic Health Science Network (AHSN).[17] The South West AHSN presented information about the study in a number of online events, and invited Trusts to email an expression of interest to the study lead to take part. Participating Trusts were offered administration costs to collect and anonymise data.

Data collection

An Excel spreadsheet (Microsoft, Redmond USA) was created to standardise the information provided by Hospital Trusts. This was piloted and refined by midwives and neonatal nurses at one of the participating Trusts, before circulating. Members of maternity and neonatal staff at each participating Trust searched their records for cases of BBA, uploaded anonymised information to the spreadsheet, and returned it to the study lead. Records were included if they related to a live birth in the prehospital setting where the baby was brought to hospital by EMS.

For peer review only - http://bmjopen.bmj.com/site/about/guidelines.xhtml

Analysis

Demographic characteristics of the mothers (booking status, parity, safeguarding status) and characteristics of the birth (gestation, temperature on admission, treatment) were reported using descriptive statistics.

Phase B: 999 call-handler advice on neonatal temperature management

Analysis of current advice

Two UK NHS EMS provider organisations (Ambulance Trusts) provided anonymised audio-recordings of calls relating to BBA which were analysed to investigate the temperature management advice given by EMS call-handlers during emergency calls.

Data collection

All EMS calls relating to BBA between January 2021 and January 2022 were searched by the Clinical Information and Records teams at the two participating EMS provider organisations. Calls were included if they related to an EMS diagnosis code of 'normal delivery' (a single live birth with no obstetric complications). Thirty calls were selected at random by the Clinical Information and Records from postcodes with varying levels of deprivation. Deprivation was determined through the lower layer super output area of the incidents, which gave an overall index of multiple deprivation (IMD) score for each call. Anonymised audio recordings of the calls were then transferred to the study lead and transcribed by a University-approved transcription company.

Analysis

Data were analysed by LG and AJ using content analysis, and findings were used to develop an audiovisual presentation to stimulate discussion during focus groups (Figure 1).

Staff and patient views

Focus groups were conducted with NHS staff and patients to investigate opportunities to improve the temperature management advice given during EMS calls.

Recruitment

A purposive sampling technique was adopted to ensure rich information about the topic.[18] Participants were eligible to take part in staff focus groups if they were a practising member of NHS staff with experience of BBA (e.g., paramedic, midwife, neonatal nurse/doctor, EMS call-handler). Participants were eligible to take part in patient focus groups if they had experienced BBA (e.g., as a parent or caller to EMS).

Recruitment adverts were circulated using social media and collaborating organisations (South West AHSN, Stillbirth and Neonatal Death Society (SANDS) charity), which included an offer of a £25 voucher for those who took part. Eligible participants were invited to contact LG to join a focus group. Potential participants were emailed a participant information sheet, privacy notice and consent form. Verbal confirmation of consent was audio-recorded at the start of each focus group. We aimed to recruit 30-40 participants, guided by information power with a narrow aim and specific study population.[19]

Data collection

Four focus groups were held via Teams (Microsoft, Redmond USA) with NHS staff, facilitated by LG. The content and structure of BBA calls were discussed in relation to their ideas on best practice temperature management advice, in response to findings from the content analysis of EMS calls, using the presentation described above (Figure 1).

For peer review only - http://bmjopen.bmj.com/site/about/guidelines.xhtml

Themes identified during these focus groups were used to create a second audiovisual presentation to facilitate focus group discussions with members of the public (Figure 1). Five focus groups were held via Teams and focused on the content and accessibility of the advice given during EMS calls, as well as the suggestions put forward by NHS staff.

Analysis

Focus groups were video-recorded and transcripts generated by Teams. Transcripts were coded and analysed by LG using thematic analysis. Findings were synthesised to create a set of recommended adaptations to the current advice given by call-handlers using EMS triage systems. These recommended adaptations were sent to focus group participants and the PPI group for final review and refinement.

RESULTS

Phase A: Neonatal hypothermia on arrival at hospital

217 babies were conveyed to the 6 hospital Trusts by the ambulance service during the above time-period (live births ≥24 weeks). There were 33 records (15%) with no neonatal arrival temperature documented.

Of those with a temperature recorded, 35% (64/184) were hypothermic on arrival at hospital. Table 1 gives a breakdown of these recorded temperatures according to World Health Organization (WHO) classifications of core body temperature for newborns.[6]

Table 1: Neonatal temperature on arrival at hospital

WHO classification	Temperature range	Number of babies (%) n = 184	Implication for outcomes/care (WHO)
Normal range	36.5 – 37.5°C	120 (65%)	N/A – normal care
Cold stress (mild hypothermia)	36 - 36.4°C	48 (26%)	Cause for concern
Moderate hypothermia	32 - 35.9°C	14 (8%)	Danger; warm baby
Severe hypothermia	<32°C	2 (1%)	Outlook grave, skilled care urgently needed

Inequalities in chances of having a BBA

Hospital data from this study were compared to South West data from the National Maternity Data Set for 2021-2022[20].

Some characteristics were more prevalent in the data for BBAs compared with local data for all births (Table 2). This included safeguarding concerns documented at booking, previous live births, and late booking (past 13 weeks).

Table 2. Comparisons made between BBA data and overall data for the South West

Characteristic		BBA data Number (%)	Overall South West data Number (%)
**Safeguarding concerns documented at booking**		**n = 217**	**n = 48900**
	Yes	47 (22%)	4180 (8%)
	No	170 (78%)	43425 (89%)
	Missing data	0 (0%)	1295 (3%)

For peer review only

Previous live birth(s)		n = 217	n = 48905
	Yes	185 (85%)	26875 (55%)
	No	32 (15%)	19125 (39%)
	Missing data	0 (0%)	2905 (6%)
**Gestation at booking***		**n = 217**	**n = 48900**
	Early booker (up to 12+6 weeks)	179 (83%)	46020 (94%)
	Delayed booker (13 to 19+6 weeks)	20 (9%)	1835 (4%)
	Late booker/unbooked (≥20 weeks)	14 (6%)	935 (2%)
	Missing data	4 (2%)	110 (0.2%)
**Mother's age at booking**		**n = 217**	**n = 48900**
	Under 20	7 (3%)	1410 (3%)
	20 to 29	90 (41%)	20,000 (41%)
	30 to 39	108 (50%)	25,725 (53%)
	40 or over	11 (5%)	1765 (4%)
	Missing data	1 (0.1%)	0 (0%)

*Booking classifications are presented as defined by The National Institute for Health and Care Excellence (NICE)[21]

Whilst we set out to compare the ethnicity of women experiencing BBA with the overall South West data, this was prevented by the use of different ethnicity categories in the two data sets. For those experiencing BBA recorded ethnicity was: British European 94%, East European 2%, Other 2%, Indian 0.5%, North European 0.5%, South African 0.5%, South European 0.5%. For those in the South West data set ethnicity was: White 84%, Asian or Asian British 3%, Black or Black British 2%, Mixed 2%, Any other ethnic group 2%.

Inequalities in neonatal hypothermia/poor outcomes

Hypothermia on arrival at hospital was more prevalent pre-term babies, babies born to mothers who had not had a previous live birth, and babies born to mothers who reported a disability or safeguarding concern at booking (Table 3).

Table 3: Characteristics of mothers and babies, by neonatal temperature on arrival at hospital, n = 184

Characteristic of baby/mother		<36.5°C n = 64 Number (%)	≥36.5°C n = 120 Number (%)
Prematurity	Pre-term	19 (30%)	20 (17%)
	Term	44 (69%)	99 (82%)
	Unknown	1 (1%)	1 (1%)
Previous live birth	Yes	48 (75%)	109 (91%)
	No	16 (25%)	11 (9%)
Disability reported at booking	Yes	6 (9%)	2 (2%)
	No	58 (91%)	116 (96%)

For peer review only

	Unknown	0 (0%)	2 (2%)
Safeguarding concerns reported at booking	Yes	19 (30%)	20 (17%)
	No	45 (70%)	100 (83%)
Mother's ethnicity	British European	58 (91%)	114 (95%)
	East European	3 (4.5%)	0 (0%)
	Indian	0 (0%)	1 (1%)
	North European	1 (1.5%)	0 (0%)
	South African	1 (1.5%)	0 (0%)
	South European	0 (0%)	1 (1%)
	Other	1 (1.5%)	4 (3%)
Mother born in UK	Yes	57 (89%)	113 (94%)
	No	6 (9%)	5 (4%)
	Unknown	1 (2%)	2 (2%)
Model of care	Consultant-led	25 (39%)	48 (40%)
	Midwifery-led	37 (57%)	71 (59%)
	Unknown	2 (3%)	1 (1%)
Mother's attendance at antenatal appointments	All attended	52 (81%)	88 (73%)
	Not all attended	12 (19%)	26 (22%)
	Unknown	0 (0%)	6 (5%)
**Characteristic of baby/mother**		**<36.5°C n = 64 Mean (SD)**	**≥36.5°C n = 120 Mean (SD)**
Mother's age at booking		28 (6.5)	31 (5.6)

Babies with neonatal hypothermia on arrival at hospital were more likely to be admitted to a Neonatal Intensive Care Unit (NICU) or receive advanced care (32.8% vs 19.2%). Seven (10.9%) hypothermic babies had an extended length of stay in hospital (>7 days) whereas none of the warm babies did. More treatment was given to hypothermic babies (glucose: 7.8% vs 0.8%; radiant heat source: 20.3% vs 3.3%; and incubator: 10.9% vs 2.5%).

Phase B: EMS call-handler advice on neonatal temperature management

Participant characteristics

Eighteen NHS staff took part in the four staff focus groups. Most participants were female (13/18, 72%). Participants' job titles comprised: midwives, paramedics, call-handlers, learning development officer, neonatal nurses, neonatal doctors, advanced care practitioners.

Twenty-two members of the public who had experience of prehospital birth took part in five focus groups. Most participants were female (18/22, 82%). Participants had a range of experiences, representing diverse geographical regions, areas of deprivation, and prehospital birth experience (e.g., at home, in the car, outside).

For peer review only

For peer review only - http://bmjopen.bmj.com/site/about/guidelines.xhtml

Five main themes were identified as affecting neonatal temperature management during EMS calls. These included: 1) the importance placed on neonatal temperature; 2) advice on where the baby should be placed following birth; 3) advice on how to keep the baby warm; 4) the timing of temperature management advice; and 5) the clarity and priority of instructions.

Theme 1: The importance placed on neonatal temperature

Although call-handlers from both triage systems repeated the instruction to 'keep mum and baby warm' at several points during the EMS call, participants noted that the importance of neonatal temperature was not explicitly addressed. This was of concern to participants who had experienced a BBA, who said that they were unaware of how quickly babies could become hypothermic in the prehospital setting, or how this could affect neonatal outcomes. It was noted by participants that antenatal information did not provide much information on neonatal hypothermia so they were unprepared for it.

> *Nothing was mentioned about keeping the baby warm and to be honest, I had no idea that that's something that I should be doing.* (Public Focus Group 2)

Most participants reported that call-handlers had focused their advice and instructions on the person giving birth (e.g., determining whether the placenta had been delivered/whether there was any bleeding), and that limited attention had been given to the baby once it was established that they were breathing. Some reported that their baby had become hypothermic following their BBA and expressed frustration that it had not been made a priority or been communicated how important it was to keep their baby warm.

> *When I delivered my daughter, their concern was mainly on delivering the placenta. It was my mum that had suggested that I should take my baby into my jumper that I was wearing to keep her warm because it was a cold morning. It was only until they realised that they couldn't deliver my placenta that they told me to get the blanket out and then wrap my daughter.* (Public Focus Group 2)

All participants felt that the importance of temperature could be further highlighted. Participants who had experienced prehospital birth suggested that this should be communicated in clear and simple language. NHS staff suggested that giving the caller the 'job' of keeping the baby warm could help emphasise this importance and increase the chance of temperature management being performed well.

> *You'll find that people want something to do. They want to focus in on something. And if you were to give that information around the importance of thermal regulation 'keeping that baby warm is really important now/ When that baby's born, we really want to keep that baby warm.' I think that would be really useful for that person who's trying to do something positive.* (Staff Focus Group 2)

Theme 2: Where the baby should be placed following birth

The two triage systems differed in their instruction on where the baby should be placed following the birth. The AMPDS triage system states that callers should 'keep the baby between the mother's legs and level with her bottom', while NHS Pathways states that callers should 'place the baby on the mother's chest or tummy'.

For peer review only

Participants expressed frustration at the instruction to place the baby between the mother's legs and felt this could be very detrimental to temperature management, especially if the mother was in a room with a cold floor, such as a bathroom or kitchen.

> *Often when we're reviewing cases, we'll kind of note that as we kind of arrived on scene, the baby is on the floor and the mother's just in shock and the babies on a cold floor and it's drafty.* (Staff Focus Group 2)

Participants who had received this advice following prehospital birth said that it had caused confusion, as it contradicted what they had been taught antenatally (i.e., skin-to-skin contact following birth).

> *What we found really really confusing is that all the way through we were advised skin-to-skin, and don't do anything with the cord - keep that connected as long as possible. But then when it came to the actual 999 caller, it was totally different advice and it was 'keep the baby on the back'…Having that totally conflicting advice put my partner in the position where he was like 'I'm totally confused'.* (Public Focus Group 4)

For those who had given birth in a car, this advice had proven especially challenging. One participant noted that by trying to keep the baby between her legs her husband had had to open all the car doors, which had potentially contributed to her baby being hypothermic on arrival at hospital.

Some participants spoke of not being 'allowed' to pick their baby up following the BBA and to keep their baby on its back on the bed/floor until the paramedics arrived.

> *When the baby did come out, she was just lying on the bed on her back. And I instinctively wanted to take her and put her on my chest like I did before with the midwives, and the instruction was to leave her on her back. I wasn't allowed to pick her up.* (Public Focus Group 4)

Skin-to-skin contact with the mother was put forward by NHS staff as the optimal practice for neonatal normothermia following prehospital birth. There was some discussion amongst staff regarding the importance of maintaining a clear airway during skin-to-skin contact, and it was acknowledged that regular prompts would need to be given by call-handlers to check that the baby's nose and mouth could be seen. Participants also emphasised the need for clear and simple instructions to be given by call-handlers on how to conduct skin-to-skin contact effectively, including checking to see if the mother was warm themselves.

Most participants said that skin-to-skin contact would have been practical in the circumstances surrounding their BBA. Some circumstances were seen as potentially problematic for skin-to-skin contact, for example, if the cord was too short, they were too anxious, or were not confident in handling the baby (e.g., first-time parents). Some participants expressed concerns over privacy regarding skin-to-skin contact and said that they would not feel confident doing this in a public place or if they thought male paramedics might attend.

> *I was in a shopping mall, and there were lots of people around me. Lots of people looking and crowding. So I couldn't feel like I could do skin-to-skin. I was in panic and the security guy was a man. I'm sad I didn't get to do it, but it was too much stress.* (Public Focus Group 2)

Staff also acknowledged that there may be cases where skin-to-skin contact was not appropriate or possible, for which staff suggested thorough drying and wrapping of the baby before placing them on someone warm.

For peer review only - http://bmjopen.bmj.com/site/about/guidelines.xhtml

Ensuring that the baby was put in the optimal place was discussed by participants as particularly important in the context of long wait times for ambulances and the "cost of living crisis". Participants noted that people could potentially be waiting for hours for an ambulance in a cold house, so ensuring the baby is kept as warm as possible should be a priority when advising callers where to place the baby following birth.

Theme 3: How to keep the baby warm

Neither of the two triage systems contain specific advice on how to keep the baby warm, although some call-handlers did add some advice themselves during calls (e.g., suggesting the use of a hat). Generic advice of 'keep mum and baby warm' was given by each pathway, and callers were also told to 'cover the baby's head, but not its face' and to 'use towels and blankets to keep the baby warm'.

During focus groups with NHS staff, participants expressed a preference for warming advice to be more specific and targeted. Suggestions included advising skin-to-skin contact (as above), putting a hat on the baby, considering possible changes to the environment (e.g., closing any windows, moving off cold floors, putting the heating on), and emphasising the need to dry the baby thoroughly and replace wet towels with dry ones. Participants also suggested adding an instruction for callers to check the warmth of the baby by touching the skin on the chest/back, to see if warming measures were working.

The specific advice put forward by staff was viewed positively in focus groups with those who had experienced a BBA. Many suggested that they had experienced stress and anxiety during their BBA, and that this had caused them to 'lose all common sense' or be unable to think rationally. As such, the instruction to 'keep the baby warm' was viewed as too generic and ambiguous by participants, who felt that clear, practical instructions would be easier to follow and would help them to feel more in control of the situation.

> *I completely did not think straight in that moment at all…I was just a bit of an idiot, to be honest. And so when he came out, I didn't think about keeping him warm…I don't think you can rely on people thinking straight in that moment. … I think sometimes like I said, stating the obvious is quite useful in that kind of scenario.* (Public Focus Group 4)

Most participants felt that all warming measures suggested by staff would have been practical in the circumstances of their BBA, and that alternatives would have been easily considered if not (e.g., using an item of clothing instead of a blanket/towel). Participants did acknowledge, however, that some warming measures may not be practical in all situations, and that callers should be advised that each warming measure should be carried out 'if possible'. Participants also noted that listing all warming measures may not be appropriate if the woman was calling for herself and had no-one around to assist her.

Of the measures suggested by staff, potential changes to the environment were emphasised by participants as something that could have considerably influenced their baby's temperature. Participants suggested that simple advice such as moving to a warmer room would have given them a sense that they were positively impacting the situation and helped relieve some of the anxiety and panic.

> *We had quite a long wait before the midwife came. My husband was really panicking…but actually if someone had said to him 'move to a warm room' or 'go and put the heating on, if you haven't got it on already', he would have done that straight away.* (Public Focus Group 4)

For peer review only - http://bmjopen.bmj.com/site/about/guidelines.xhtml

Theme 4: Timing of temperature management advice

Another key theme about the content and structure of the EMS triage systems was the timing of neonatal temperature management advice. While call-handlers from both pathways focused this advice predominantly after the baby was born, long gaps were identified in some calls while call-handlers waited for a part of the baby to be seen by the caller. NHS staff suggested that these gaps could be used to emphasise the importance of temperature, and to give advice on warming the environment if appropriate. Gaps in the conversation were also identified during periods of time where call-handlers had completed the triage algorithm and were waiting for paramedics to arrive. These gaps were often filled with small-talk. Staff suggested that this would be an appropriate time to give specific warming advice, and for call-handlers to check that essential warming measures had been completed.

> *The gap should be used to paraphrase back to them. Just saying, you know…like 'dry, warm and wrapped'. Just keeping it simple repeating it back to them, showing that they understand the instruction for…however long it takes [the paramedics] to finally get there.* (Staff Focus Group 1)

Participants who had experienced a BBA felt that warming advice should be given as soon as possible, but without interrupting vital instructions. Some participants felt that the woman should be the priority and focus of calls before the baby was born, but that a brief mention of the importance of temperature would be helpful, and that focus should then shift to keeping the baby warm after birth.

> *If it seems like it's a rushed labour, then perhaps maybe focus on what's going on at hand. And then perhaps maybe give the [temperature] advice thereafter like step by step... Whereas if it's maybe not as quick, maybe strike some sort of conversation with the person that's with the mother and say 'ok, when the baby does come out, remember to keep the baby warm.* (Public Focus Group 4)

Participants agreed with NHS staff that once the triage algorithm was complete, any additional time spent on calls waiting for the ambulance should be used to provide specific warming advice and to check it had been followed appropriately. Participants felt that it would be helpful to be reminded of all the steps they could take to keep their baby warm at any possible opportunity.

> *If they can [provide warming advice] straight away if they've got time, then yeah, do it again also in between [gaps in the conversation]. If they've got time waiting for the ambulance, just mention it again. Just maybe check, check baby's chest or, you know, make sure it's warm or just little things like that because you do, you do forget.* (Public Focus Group 1)

Focus group participants also discussed advice that could be given antenatally to prepare them for neonatal temperature management in the prehospital setting. Suggestions included an antenatal leaflet on the importance of neonatal thermoregulation, a reminder at antenatal appointments, and/or advice to pack a blanket in their hospital bag (in case of birthing in the car).

Theme 5: Clarity and priority of instructions

The clarity and priority given to instructions was discussed by participants in both focus groups. Several instructions were highlighted by participants as potentially confusing or distracting, and some felt that the timing and priority given to these instructions could delay neonatal temperature management. For example, calls from the NHS Pathways algorithm included instructions for the caller to 'wipe or peel away the sac' before drying and wrapping the baby. This frequently caused confusion to callers, as none of the babies were born in their sac and meant that drying and

For peer review only

wrapping instructions were delayed while the call-handler and caller discussed the nature of the sac. NHS staff suggested that the previous instruction to 'wipe the baby's nose and mouth' should be sufficient to cover any issues with the sac and suggested that this instruction be removed or altered to avoid confusion. Participants from both sets of focus groups suggested that once the baby was born, only essential instructions should be given before warming advice, as to avoid any confusion or distraction.

> *Babies being born in the sac is really rare. Usually it's you know, contractions, suddenly the water's gone, and the baby follows it quite quickly with these mums…So I don't think that should even be in there really because that might even worry mums like 'well, should there be a sac? Is it wrong that they're not in a sac? Oh my goodness me, you know, where's the sac?' And then start looking for a sac. (Staff Focus Group 4)*

Other instructions questioned by NHS staff included the advice to get towels and blankets to 'protect the bed or floor', and the caller being directed to wash their hands once a part of the baby could be seen.

DISCUSSION

This is the first study, to our knowledge, to investigate the temperatures of babies arriving at hospital following a prehospital birth (birth before arrival; BBA) in South West England. We found that 35% of babies born prehospital arrived at hospital hypothermic (<36.5°C), and 25% of these were classed as 'moderately' or 'severely' hypothermic, with warming needed urgently to prevent poor outcomes. These findings are in line with those of a study in Finland, which found hospital diagnosis of neonatal hypothermia to be recorded in 53% of babies born out of hospital, compared to only 0.7% of babies born in hospital.[2] Our findings also concur with research from France, which found that 56% of babies born outside of hospital were hypothermic on arrival at hospital,[15] and that neonatal hypothermia was associated with increased rates of NICU admission.[3]

We found that particular babies may be more at risk of hypothermia on hospital arrival following BBA. These include pre-term babies, babies of first-time mums, babies of women with safeguarding concerns, and babies of women who reported disabilities at their booking appointment. There is a paucity of previous literature in this area, and our study is limited by the small sample size and focused geographical location. However, these preliminary associations provide a basis for future research, and may help NHS Trusts identify babies who require additional warming and support following a BBA.

This study is the first to investigate neonatal temperature management advice provided by EMS call-handling triage systems. Our findings suggest five themes that could influence neonatal thermal care in the advice currently scripted for UK EMS calls regarding BBA. NHS staff and members of the public made several recommendations for changes to the script to address potential barriers to good thermal care, based on these themes. Although this is the first study to investigate neonatal temperature management advice given by EMS call-handlers, increasing attention is being paid to call-handler advice and interactions with patients,[22] especially during maternity cases.[16]

Finally, our findings suggest that there may be some women in South West England who are more likely to experience BBA, which previous research suggests may result in poorer outcomes for their babies.[2,3] These include women with safeguarding concerns, women who book their pregnancies with a health professional later than 13 weeks, and those who have had babies before. Multiparity, late booking and/or lack of antenatal care are known to be associated with BBA from previous

For peer review only - http://bmjopen.bmj.com/site/about/guidelines.xhtml

literature,[2,10,11,23] however safeguarding concerns at booking has not been identified previously. Due to the small sample size of this study, caution should be taken when interpreting results but this finding may help maternity services identify and support women who are more likely to experience BBA. Despite receiving information on mothers' ethnicity from our BBA data, we were unable to explore any associations, as local ethnicity data were recorded differently to the National Maternity Data Set.[20] Work is ongoing to resolve this issue.

CONCLUSION

The findings of this study suggest there may be certain women who are more likely to have a BBA in South West England and certain babies who are more likely to be hypothermic on arrival at hospital following a BBA. Findings also suggest potential areas for improvement to EMS call-handler advice regarding neonatal temperature management. Future work in this area has the potential to reduce the number of babies arriving at hospital hypothermic and improve longer term outcomes, thereby addressing health inequalities by disproportionately benefitting underserved groups.

LEGENDS

Figure 1: Flowchart representing the process of data collection and analysis.

STATEMENTS

Acknowledgments

We would like to thank the NHS staff and members of the public who took part in focus groups for this evaluation, as well as the NHS Hospital and Ambulance Service Trusts who provided data.

We would also like to thank: Alice Jennings from UWE Bristol, for supporting the collection of hospital data and contributing to analysis of EMS calls; Sally Hedge from the South West AHSN for supporting and promoting this work; and the members of our Patient and Public Involvement Group for their invaluable input and contributions to this study: Tasha Cooper, Farzana Kausir, Poonam Barnes, Louise Walker, Simon Walker and Arif Hoque.

Finally, we would like to thank the funder of this project, the South West AHSN, and the study sponsor, UWE Bristol. This work was carried out by members of the Research in Emergency Care, Avon Collaborative Hub (REACH Bristol).

Patient and Public Involvement

Patients and/or the public were involved in the design, or conduct, or reporting, or dissemination plans of this research. Refer to the Methods section for further details.

Patient consent for publication

Not required

Ethics committee approval

For peer review only - http://bmjopen.bmj.com/site/about/guidelines.xhtml

For peer review only

Ethics committee approval for this service evaluation was granted by the University of the West of England (UWE Bristol) Faculty of Health and Applied Sciences Research Ethics Committee (HAS.22.01.061). Participants gave informed consent to participate in the study before taking part.

Provenance and peer review

Not commissioned; externally peer reviewed.

Contributorship statement

LG was the Chief Investigator/guarantor with overall responsibility for the study, and participated in study conception, design and coordination, performed and coded the focus groups, analysed the data and drafted the manuscript. HT participated in study conception, design and interpretation of the results, and supported quantitative data analysis. GM, KK, EB, AB, TD, RO, HM, RMC, NM, SV and JB participated in study conception, design and interpretation of the results. All authors were responsible for the critical revision of the manuscript for publication and approved the final version to be published.

Competing interests

None declared

Funding

The study was funded by the South West Academic Health Science Network (AHSN) through its Perinatal Equity programme.

Data sharing statement

Data are available upon reasonable request.

REFERENCES

1. Snowden JM, Tilden EL, Snyder J, et al. Planned out-of-hospital birth and birth outcomes. *New England Journal of Medicine*. 2015;373(27):2642-2653.

2. Ovaskainen K, Ojala R, Gissler M, et al. Out-of-hospital deliveries have risen involving greater neonatal morbidity: risk factors in out-of-hospital deliveries in one University Hospital region in Finland. *Acta Paediatr*. 2015;104(12):1248-1252.

3. Javaudin F, Hamel V, Legrand A, et al. Unplanned out-of-hospital birth and risk factors of adverse perinatal outcome: findings from a prospective cohort. *Scand J Trauma Resusc Emerg Med*. 2019;27(1):26.

4. Nguyen ML, Lefevre P, Dreyfus M. Maternal and neonatal outcomes of unplanned deliveries. *J Gynecol Obstet Biol Reprod (Paris)*. 2016;45(1):86-91. doi:10.1016/j.jgyn.2015.02.002

5. Chitty H, Wyllie J. Importance of maintaining the newly born temperature in the normal range from delivery to admission. *Seminars in Fetal and Neonatal Medicine*. 2018; 18(6):362-368.

6. World Health Organization. *Thermal Control of the Newborn: A Practical Guide* [online]. 1993. https://apps.who.int/iris/bitstream/handle/10665/60042/WHO_FHE_MSM_93.2.pdf (accessed March 13, 2023).

For peer review only - http://bmjopen.bmj.com/site/about/guidelines.xhtml

7. Perlman JM, Wyllie J, Kattwinkel J, et al. Part 7: neonatal resuscitation: 2015 international consensus on cardiopulmonary resuscitation and emergency cardiovascular care science with treatment recommendations. *Circulation*. 2015;132(16,1):S204-S241.

8. Goodwin L, Voss S, McClelland G, et al. Temperature measurement of babies born in the pre-hospital setting: analysis of ambulance service data and qualitative interviews with paramedics. *Emergency Medicine Journal*. 2022;39(11):826-832.

9. McClelland G, Burrow E, McAdam H. Babies born in the pre-hospital setting attended by ambulance clinicians in the north east of England. *Br Paramed J*. 2019;4(3):43-48.

10. Loughney A, Collis R, Dastgir S. Birth before arrival at delivery suite: associations and consequences. *Br J Midwifery*. 2006;14(4):204-208.

11. Renesme L, Garlantézec R, Anouilh F, et al. Accidental out-of-hospital deliveries: a case–control study. *Acta Paediatr*. 2013;102(4):174-177.

12. Diana L, Glaucia L, Adriana C, et al. Out-of-hospital deliveries: A case-control study. *Turkish Archives of Pediatrics.* 2018;53(2):87.

13. Moscovitz HC, Magriples U, Keissling M, et al. Care and Outcome of Out-of-hospital Deliveries. *Academic Emergency Medicine*. 2000;7(7):757-761.

14. Adamsons K, Towell ME. Thermal homeostasis in the fetus and newborn. *The Journal of the American Society of Anesthesiologists*. 1965;26(4):531-548.

15. Javaudin F, Roche M, Trutt L, et al. Assessment of rewarming methods in unplanned out-of-hospital births from a prospective cohort. *Scand J Trauma Resusc Emerg Med*. 2020;28:1-9.

16. Healthcare Safety Investigation Branch. *Maternity Pre-Arrival Instructions by 999 Call Handlers. Independent Report by the Healthcare Safety Investigation Branch NI-000818* [online]. 2022. https://hsib-kqcco125-media.s3.amazonaws.com/assets/documents/hsib-report-maternity-pre-arrival-instructions-by-999-call-handlers.pdf (accessed August 21, 2023).

17. South West Academic Health Network. *SW AHSN* [online]. 2022. https://swahsn.com/ (accessed September 21, 2023).

18. Patton MQ. *Qualitative Research & Evaluation Methods: Integrating Theory and Practice*. Sage publications; 2014.

19. Malterud K, Siersma VD, Guassora AD. Sample size in qualitative interview studies: guided by information power. *Qual Health Res*. 2016;26(13):1753-1760.

20. NHS Digital. *NHS Maternity Statistics, 2021-2022: MSDS CSV Data* [online]. 2022. https://digital.nhs.uk/data-and-information/publications/statistical/nhs-maternity-statistics/2021-22 (accessed March 13, 2023).

21. National Collaborating Centre for Women's and Children's Health. *NICE Clinical Guideline: Pregnancy and complex social factors: a model for service provision for pregnant women with complex social factors* [online]. 2010. https://www.nice.org.uk/Guidance/CG110 (accessed May 20, 2023).

For peer review only - http://bmjopen.bmj.com/site/about/guidelines.xhtml

22. Perera N, Finn J, Bray J. Can emergency dispatch communication research go deeper? *Resusc Plus*. 2022;9. doi: 10.1016/j.resplu.2021.100192

23. Bhoopalam S, Watkinson M. Babies born before arrival at hospital. *BJOG*. 1991;98(1):57-64.

For peer review only

For peer review only - http://bmjopen.bmj.com/site/about/guidelines.xhtml

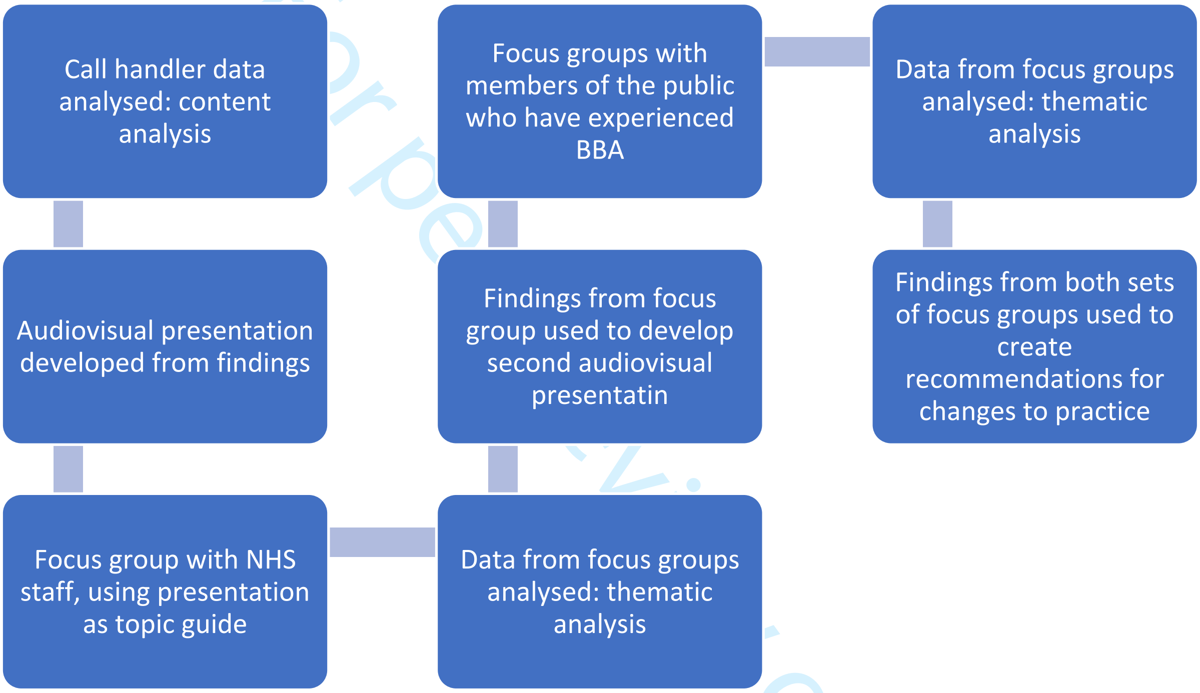

Figure 1: Flowchart representing the process of data collection and analysis.

Supplementary Table 1: COREQ checklist

**Domain 1: Research team and reflexivity**		**Location in manuscript (Section, page no.)**
**Personal Characteristics**		
1. Interviewer/facilitator Which author/s conducted the interview or focus group?	LG	Methods - 5
2. Credentials<br>What were the researcher's credentials? E.g. PhD, MD	BSc, PhD	Title Page
3. Occupation<br>What was their occupation at the time of the study?	Senior Research Fellow in Emergency Care	Title Page
4. Gender Was the researcher male or female?	Female	N/A
5. Experience and training<br>What experience or training did the researcher have?	Experienced qualitative researcher.	Methods - 5
**Relationship with participants**		
6. Relationship established<br>Was a relationship established prior to study commencement?	No	N/A
7. Participant knowledge of the interviewer<br>What did the participants know about the researcher? e.g. personal goals, reasons for doing the research	Participants were briefed on the purpose of the study and were aware that ethical approval had been granted. Participants reviewed the participant information documentation prior to giving their written informed consent to be involved.	Methods - 5
8. Interviewer characteristics What characteristics were reported about the interviewer/facilitator? e.g. Bias, assumptions, reasons and interests in the research topic	No interviewer-related biases identified.	N/A
**Domain 2: study design**		
**Theoretical framework**		
9. Methodological orientation and Theory<br>What methodological orientation was stated to underpin the study? e.g. grounded theory, discourse analysis, ethnography, phenomenology, content analysis	Thematic analysis	Methods – 5
Participant selection		
10. Sampling How were participants selected? e.g.	Purposive sampling was used: recruitment adverts were circulated using social media and collaborating organisations	Methods - 5

For peer review only - http://bmjopen.bmj.com/site/about/guidelines.xhtml

11. Method of approach How were participants approached? e.g. face-to-face, telephone, mail, email	As above	Methods - 5
12. Sample size How many participants were in the study?	18 NHS staff and 22 members of the public	Results - 7
13. Non-participation How many people refused to participate or dropped out? Reasons?	There were no participants who subsequently refused to participate, withdrew consent or dropped out.	N/A
**Setting**		
14. Setting of data collection Where was the data collected? e.g. home, clinic, workplace	Data was collected and recorded via a Microsoft Teams meeting.	Methods - 5
15. Presence of non-participants Was anyone else present besides the participants and researchers?	No	N/A
16. Description of sample What are the important characteristics of the sample? e.g. demographic data, date	Most participants were female (72% of the NHS staff and 82% of the patients). For NHS staff, participants' job titles comprised: midwives, paramedics, call-handlers, learning development officer, neonatal nurses, neonatal doctors, advance care practitioners. For members of the public, participants had a range of experiences, representing diverse geographical regions, areas of deprivation, and prehospital birth experience (e.g., at home, in the car, outside).	Results – 7
**Data collection**		

For peer review only

17. Interview guide Were questions, prompts, guides provided by the authors? Was it pilot tested?	Focus group discussions were semi-structured using a PowerPoint presentation to guide discussion; the presentations were reviewed by the study team and Patient and Public Involvement group before focus groups took part.	Methods - 5
18. Repeat interviews<br>Were repeat interviews carried out? If yes, how many?	No	N/A
19. Audio/visual recording<br>Did the research use audio or visual recording to collect the data?	Focus groups were recorded Microsoft Teams.	Methods - 5
20. Field notes<br>Were field notes made during and/or after the interview or focus group?	No additional field notes were made.	N/A
21. Duration What was the duration of the interviews or focus group?	The focus groups lasted an average of 60 minutes.	N/A
22. Data saturation<br>Was data saturation discussed?	We used information power	Methods - 5
23. Transcripts returned Were transcripts returned to participants for comment and/or correction?	No	N/A
**Domain 3: analysis and findings**		
**Data analysis**		
24. Number of data coders<br>How many data coders coded the data?	One - LG	Methods - 5
25. Description of the coding tree<br>Did authors provide a description of the coding tree?	N/A – not used	N/A
26. Derivation of themes<br>Were themes identified in advance or derived from the data?	Themes were derived from the data	Methods - 5
27. Software What software, if applicable, was used to manage the data?	N/A	N/A
28. Participant checking<br>Did participants provide feedback on the findings?	No	N/A
**Reporting**		
29. Quotations presented Were participant quotations presented to illustrate the themes / findings? Was each<br>quotation identified? e.g. participant number	Yes	Results – 7-11

For peer review only

30. Data and findings consistent<br>Was there consistency between the data presented and the findings?	Yes	N/A
31. Clarity of major themes<br>Were major themes clearly presented in the findings?	Yes	Results 7-11
32. Clarity of minor themes<br>Is there a description of diverse cases or discussion of minor themes?	Yes	Results 7-11

For peer review only

BMJ Open

Inequalities in birth before arrival at hospital in South West England: a multi-methods study of neonatal hypothermia and Emergency Medical Services call-handler advice

Journal:	*BMJ Open*
Manuscript ID	bmjopen-2023-081106.R1
Article Type:	Original research
Date Submitted by the Author:	01-Feb-2024
Complete List of Authors:	Goodwin, Laura; University of the West of England, School of Health and Social Wellbeing<br>Kirby, Kim; South Western Ambulance Service NHS Foundation Trust, Research, Audit and Quality Improvement Department; University of the West of England, School of Health and Social Wellbeing<br>McClelland, Graham; Northumbria University<br>Beach, Emily; University of the West of England, School of Health and Social Wellbeing<br>Bedson, Adam; South Western Ambulance Service NHS Foundation Trust<br>Benger, Jonathan; University of the West of England, School of Health and Social Wellbeing<br>Deave, Toity; University of the West of England, Centre for Child and Adolescent Health<br>Osborne, Ria; South Western Ambulance Service NHS Foundation Trust, Research, Audit & Improvement<br>McAdam, Helen; University of Sunderland, Health Sciences and Wellbeing<br>McKeon-Carter, Roisin; University Hospitals Plymouth NHS Trust<br>Miller, Nick; University of the West of England, School of Health and Social Wellbeing<br>Taylor, Hazel; University Hospitals Bristol NHS Foundation Trust, Research Design Service<br>Voss, Sarah; University of the West of England, School of Health and Social Wellbeing
**Primary Subject Heading**:	Emergency medicine
Secondary Subject Heading:	Obstetrics and gynaecology, Health services research, Paediatrics
Keywords:	ACCIDENT & EMERGENCY MEDICINE, Health Equity, NEONATOLOGY, Maternal medicine < OBSTETRICS, Paediatric A&E and ambulatory care < PAEDIATRICS

SCHOLARONE™
Manuscripts

For peer review only - http://bmjopen.bmj.com/site/about/guidelines.xhtml

I, the Submitting Author has the right to grant and does grant on behalf of all authors of the Work (as defined in the below author licence), an exclusive licence and/or a non-exclusive licence for contributions from authors who are: i) UK Crown employees; ii) where BMJ has agreed a CC-BY licence shall apply, and/or iii) in accordance with the terms applicable for US Federal Government officers or employees acting as part of their official duties; on a worldwide, perpetual, irrevocable, royalty-free basis to BMJ Publishing Group Ltd ("BMJ") its licensees and where the relevant Journal is co-owned by BMJ to the co-owners of the Journal, to publish the Work in this journal and any other BMJ products and to exploit all rights, as set out in our licence.

The Submitting Author accepts and understands that any supply made under these terms is made by BMJ to the Submitting Author unless you are acting as an employee on behalf of your employer or a postgraduate student of an affiliated institution which is paying any applicable article publishing charge ("APC") for Open Access articles. Where the Submitting Author wishes to make the Work available on an Open Access basis (and intends to pay the relevant APC), the terms of reuse of such Open Access shall be governed by a Creative Commons licence – details of these licences and which Creative Commons licence will apply to this Work are set out in our licence referred to above.

Other than as permitted in any relevant BMJ Author's Self Archiving Policies, I confirm this Work has not been accepted for publication elsewhere, is not being considered for publication elsewhere and does not duplicate material already published. I confirm all authors consent to publication of this Work and authorise the granting of this licence.

For peer review only

Inequalities in birth before arrival at hospital in South West England: a multi-methods study of neonatal hypothermia and Emergency Medical Services call-handler advice

Authors:
Goodwin, L.[1], Kirby, K.[1,2], McClelland, G.[3], Beach, E.[1], Bedson, A.[3], Benger, J.[1], Deave, T.[1], Osborne, R.[3], McAdam, H.[4], McKeon-Carter, R.[5], Miller, N.[1], Taylor, H.[6], & Voss, S.[1]

Institutions:
[1] University of the West of England, Bristol
[2] South Western Ambulance Service NHS Foundation Trust
[3] Northumbria University
[4] University of Sunderland
[5] University Hospitals Plymouth NHS Trust
[6] University Hospitals Bristol and Weston NHS Foundation Trust

Corresponding author:
Kim Kirby – Kim.Kirby@uwe.ac.uk
Keywords: Infant, Newborn, Ambulances, Hypothermia, Temperature, Emergency Service, Hospital

List of abbreviations:
AHSN – Academic Health Science Network
BBA – Birth Before Arrival at hospital
PPI – Patient and Public Involvement
SWASFT – South Western Ambulance Service NHS Foundation Trust
UK – United Kingdom

Word count: 4984

For peer review only

ABSTRACT

Objectives: To examine inequalities in birth before arrival (BBA) at hospital in South West England and understand which groups are most likely to experience BBA, and how this relates to hypothermia and outcomes (Phase A). To investigate opportunities to improve temperature management advice given by Emergency Medical Services (EMS) call-handlers during emergency calls regarding BBA in the United Kingdom (Phase B).

Design: A two-phase multi-method study. Phase A analysed anonymised data from hospital neonatal records between January 2018 and January 2021. Phase B analysed anonymised EMS call transcripts, followed by focus groups with National Health Service (NHS) staff and patients.

Setting: Six Hospital Trusts in South West England, and two EMS providers (ambulance services) in South West and North East England.

Participants: Eighteen multidisciplinary NHS staff and 22 members of the public who had experienced BBA in the UK.

Results: 35% (64/184) of babies conveyed to hospital were hypothermic on arrival. When compared with national data on all births in the South West, we found higher percentages of women with documented safeguarding concerns at booking, previous live births, and 'late bookers' (booking their pregnancy >13 weeks gestation). These women may therefore be more likely to experience BBA. Pre-term babies, babies to first-time mothers, and babies born to mothers with disability or safeguarding concerns at booking were more likely to be hypothermic following BBA. Five main themes emerged from qualitative data on call-handler advice: 1) the importance placed on neonatal temperature; 2) advice on where the baby should be placed following birth; 3) advice on how to keep the baby warm; 4) the timing of temperature management advice; 5) the clarity and priority of instructions.

Conclusions: Findings identified factors associated with BBA and neonatal hypothermia following BBA. Improvements to EMS call-handler advice could reduce the number of babies arriving at hospital hypothermic.

STRENGTHS AND LIMITATIONS OF THIS STUDY

- To the best of our knowledge, this is the first study to investigate the temperature of babies arriving at hospital following a prehospital birth in South West England.
- This is the first study to investigate neonatal temperature management advice provided by emergency service call-handling triage systems.
- The service evaluation of hospital neonatal records had a small sample size and was confined to one geographical area which limits the representativeness of our findings.

FUNDING STATEMENT

The study was funded by the South West Academic Health Science Network as part of its Perinatal Health Equity programme.

COMPETING INTERESTS

None declared

For peer review only - http://bmjopen.bmj.com/site/about/guidelines.xhtml

BACKGROUND

In the UK, an estimated 3,700 births a year are unplanned out of hospital births, or Births Before Arrival at hospital (BBA).(1,2) These births are attended by ambulance staff such as paramedics, emergency care assistants and ambulance technicians, usually without the attendance of a qualified midwife or obstetrician. BBA is associated with unfavourable perinatal outcomes and increased mortality.(3–5)

An important risk factor for mortality following BBA is hypothermia,(5–7) which is defined by the World Health Organization (WHO) as a temperature below 36.5°C.(8) The mortality risk from hypothermia is especially prominent in premature babies; in low birth weight infants, mortality increases by 28% per 1°C decrease in admission temperature below 36.5°C.(9) Paramedics are usually the first on scene at BBAs, however recent audits of Emergency Medical Service (EMS) data in the UK suggest that paramedics do not routinely take newborn temperatures (temperatures recorded in 2-10% of cases).(10,11) When temperatures have been recorded, the majority have indicated neonatal hypothermia (72%).(10,11) Currently there are no published data to show the percentage of babies who are hypothermic on arrival to hospital following a BBA, and the resulting outcomes for these babies.

In order to prevent neonatal hypothermia and poor outcomes following BBA it is important to understand which women are most likely to experience BBA, which babies are most likely to be cold on arrival, and how this interacts with outcomes such as mortality, length of hospital stay, and the need for special care and interventions. Literature around inequalities in maternal and perinatal outcomes suggests an increased risk of poor outcomes for women who do not engage or have little engagement with maternity services.(2,12) This includes concealed or teenage pregnancies, as well as women from migrant or minority ethnic backgrounds.(13,14) A call to EMS regarding labour or birth may, therefore, be the first point of contact that some women will have regarding their pregnancy.

In the prehospital setting, babies can become hypothermic within minutes(15) and are sometimes born before EMS arrival. In these instances, the newborn is more likely to be hypothermic on arrival at hospital, potentially due to ineffective warming by non-professionals.(16) Therefore, the earlier that effective warming measures can be initiated (for example at the point of calls to EMS), the better the chances of improved outcomes following BBA. A recent Healthcare Safety Investigation Branch (HSIB) report(17) highlighted the fact that UK investigations into poor patient outcomes following BBA do not routinely consider the clinical impact of advice given by EMS call-handlers, and recommended work with relevant stakeholders to develop guidance for maternity emergencies during these calls. It is therefore necessary to investigate the advice given regarding neonatal temperature management during EMS calls relating to BBA, and work with relevant stakeholders to investigate potential ways to develop and improve this advice.(17)

The aims of this project were:

1) To examine inequalities in BBA in South West England and understand which groups are most likely to experience BBA, and also how this relates to hypothermia and neonatal outcomes (Phase A).
2) To investigate opportunities to improve the temperature management advice given by EMS call-handlers during emergency calls regarding BBA in the UK (Phase B).

For peer review only - http://bmjopen.bmj.com/site/about/guidelines.xhtml

METHODS

Setting

Hospital data were collected from six NHS Hospital Trusts in South West England. The area covered by these Trusts has one of the lowest population densities in the country, with people living across a mix of coastal communities, isolated rural areas, and urban centres.(18)

Data for EMS calls were collected from two NHS ambulance service Trusts using the two different Clinical Decision Support Systems used in the UK: the Advanced Medical Priority Dispatch System (AMPDS) (Salt Lake City, USA) and NHS Pathways (NHS Digital, Leeds, UK). Data from calls triaged by AMPDS were collected from the South Western Ambulance Service NHS Foundation Trust (SWASFT), while data from calls triaged by NHS Pathways were collected from the North East Ambulance Service NHS Foundation Trust (NEAS). AMPDS has been in use in the United States since 1978 and is used by emergency services in 59 countries, including roughly half of ambulance service Trusts in the UK.(17) NHS Pathways is used by the other UK ambulance trusts and is overseen by a National Clinical Governance Group (NCGG), which is hosted by the Royal College of General Practitioners and is made up of representatives from UK medical royal colleges.(17)

Patient and public involvement (PPI)

A dedicated PPI group guided this work from its inception, supporting the development of research questions and study design. The group consisted of four women and two men who had experience of BBA. Experiences were varied and represented ethnic, cultural and geographical diversity. Over the course of the project, the PPI group reviewed and refined participant facing documents for stakeholder focus groups, contributed to data synthesis, and advised the team on the approach to recruitment and dissemination.

Study design

This multi-methods study consisted of both qualitative and quantitative data analyses, with two phases. Phase A included an analysis of hospital data and Phase B included content analysis of EMS calls, followed by thematic analysis of focus groups with NHS staff and patients.

The two phases of this study were funded as one piece of work and linked through their focus on inequality. Whilst we wanted to use Phase A of this work to examine potential inequalities surrounding BBA births in the South West of England, we also wanted to examine the accessibility of EMS call-handler advice regarding neonatal hypothermia in order to identify potential opportunities to improve this advice by ensuring we were aware of the populations most at risk, and providing them with accessible and easily understandable information.

Phase A: Neonatal hypothermia on arrival at hospital

Anonymised extracts from routinely collected non-identifiable data (hospital neonatal records for BBA babies) were provided by participating Trusts from a three-year period (January 2018-January 2021). These were compared with National Maternity Data from the same geographical area to understand which groups are most likely to experience BBA and how this relates to hypothermia and neonatal outcomes recorded by Hospital Trusts (including admission to a neonatal unit (NNU), length of hospital stay, and treatment provided).

Recruitment

Hospital Trusts were eligible to take part if they were covered by the South West Academic Health Science Network (AHSN).(18) The South West AHSN presented information about the study in a number of online events, and invited Trusts to email an expression of interest to the study lead to take part. Participating Trusts were offered administration costs to collect and anonymise data.

For peer review only - http://bmjopen.bmj.com/site/about/guidelines.xhtml

Data collection

An Excel spreadsheet (Microsoft, Redmond USA) was created to standardise the information provided by Hospital Trusts. Data points selected for inclusion were all demographic characteristics for the mother and baby, and all care and outcome information related to neonatal temperature, that were available from the maternity notes held by the Hospital Trusts. The spreadsheet was piloted and refined by midwives and neonatal nurses at one of the participating Trusts, before circulating. Members of maternity and neonatal staff at each participating Trust searched their records for cases of BBA, uploaded anonymised information to the spreadsheet, and returned it to the study lead. Records were included if they related to a live birth in the prehospital setting where the baby was brought to hospital by EMS (ambulance staff).

Analysis

Due to the small number of BBA cases recorded over the time period specified above, we did not do an in-depth statistical analysis with statistical testing. As such, demographic characteristics of the mothers (booking status, parity, safeguarding status) and characteristics of the birth (gestation, temperature on admission, treatment) were reported using descriptive statistics.

Phase B: 999 call-handler advice on neonatal temperature management

Analysis of current advice

Two UK NHS EMS provider organisations (Ambulance Trusts) provided anonymised audio-recordings of calls relating to BBA which were analysed to investigate the temperature management advice given by EMS call-handlers during emergency calls.

Data collection

All EMS calls relating to BBA between January 2021 and January 2022 were searched by the Clinical Information and Records (CIR) teams at the two participating EMS provider organisations (ambulance services). The team used an EMS diagnosis code of ‘normal delivery’ (a single live birth with no obstetric complications) to find relevant calls. Calls were listened to by the CIR team to check eligibility, and 30 eligible calls (15 from each ambulance service) were selected at random from postcodes with varying levels of deprivation (Figure 1). Deprivation was determined by the team through the lower layer super output area of the incidents, which gave an overall index of multiple deprivation (IMD) score for each call. Anonymised audio recordings of the calls were then transferred to the study lead and transcribed by a University-approved transcription company.

Analysis

Data were analysed by LG and AJ using content analysis, and findings were used to develop an audiovisual presentation to stimulate discussion during focus groups (Figure 1).

Staff and patient views

Focus groups were conducted with NHS staff and patients to investigate opportunities to improve the temperature management advice given during EMS calls.

Recruitment

A purposive sampling technique was adopted to ensure rich information about the topic.(19) Participants were eligible to take part in staff focus groups if they were a practising member of NHS staff with experience of BBA (e.g., paramedic, midwife, neonatal nurse/doctor, EMS call-handler). Participants were eligible to take part in patient focus groups if they had experienced BBA (e.g., as a parent or caller to EMS).

Recruitment adverts were circulated using social media and collaborating organisations (South West AHSN, Stillbirth and Neonatal Death Society (SANDS) charity), and participants were reimbursed with

For peer review only - http://bmjopen.bmj.com/site/about/guidelines.xhtml

a £25 e-voucher for their time. Eligible participants were invited to contact LG to join a focus group. Potential participants were emailed a participant information sheet, privacy notice and consent form. Verbal confirmation of consent was audio-recorded at the start of each focus group. We aimed to recruit 30-40 participants, guided by information power with a narrow aim and specific study population.(20)

Data collection

Four focus groups were held via Teams (Microsoft, Redmond USA) with NHS staff, facilitated by LG. The content and structure of BBA calls were discussed in relation to their ideas on best practice temperature management advice, in response to findings from the content analysis of EMS calls, using the presentation described above (Figure 1).

Themes identified during these focus groups were used to create a second audiovisual presentation to facilitate focus group discussions with members of the public (Figure 1). Five focus groups were held via Teams and focused on the content and accessibility of the advice given during EMS calls, as well as the suggestions put forward by NHS staff.

Analysis

Focus groups were video-recorded and transcripts generated by Teams. Transcripts were coded and analysed by LG using thematic analysis. Findings were synthesised to create a set of recommended adaptations to the current advice given by call-handlers using EMS triage systems. These recommended adaptations were sent to focus group participants and the PPI group for final review and refinement.

RESULTS

Phase A: Neonatal hypothermia on arrival at hospital

217 babies were conveyed to the 6 hospital Trusts by the ambulance service during the above time-period (live births ≥24 weeks). There were 33 records (15%) with no neonatal arrival temperature documented.

Of those with a temperature recorded, 35% (64/184) were hypothermic on arrival at hospital. Table 1 gives a breakdown of these recorded temperatures according to World Health Organization (WHO) classifications of core body temperature for newborns.(8)

Table 1: Neonatal temperature on arrival at hospital

WHO classification	Temperature range	Number of babies (%) n = 184	Implication for outcomes/care (WHO)
Normal range	36.5 – 37.5°C	120 (65%)	N/A – normal care
Cold stress (mild hypothermia)	36 - 36.4°C	48 (26%)	Cause for concern
Moderate hypothermia	32 - 35.9°C	14 (8%)	Danger; warm baby
Severe hypothermia	<32°C	2 (1%)	Outlook grave, skilled care urgently needed

Inequalities in chances of having a BBA

Hospital data from this study were compared to South West data from the National Maternity Data Set for 2021-2022(21).

Some characteristics were more prevalent in the data for BBAs compared with local data for all births (Table 2). This included safeguarding concerns documented at booking, previous live births,

For peer review only - http://bmjopen.bmj.com/site/about/guidelines.xhtml

and late booking (past 13 weeks). A definition of all characteristics are provided in Supplemental file 1.

Table 2. Comparisons made between BBA data and overall data for the South West

Characteristic		BBA data Number (%)	Overall South West data Number (%)
**Safeguarding concerns documented at booking**		**n = 217**	**n = 48900**
	Yes	47 (22%)	4180 (8%)
	No	170 (78%)	43425 (89%)
	Missing data	0 (0%)	1295 (3%)
**Previous live birth(s)**		**n = 217**	**n = 48905**
	Yes	185 (85%)	26875 (55%)
	No	32 (15%)	19125 (39%)
	Missing data	0 (0%)	2905 (6%)
**Gestation at booking***		**n = 217**	**n = 48900**
	Early booker (up to 12+6 weeks)	179 (83%)	46020 (94%)
	Delayed booker (13 to 19+6 weeks)	20 (9%)	1835 (4%)
	Late booker/unbooked (≥20 weeks)	14 (6%)	935 (2%)
	Missing data	4 (2%)	110 (0.2%)
**Mother's age at booking**		**n = 217**	**n = 48900**
	Under 20	7 (3%)	1410 (3%)
	20 to 29	90 (41%)	20,000 (41%)
	30 to 39	108 (50%)	25,725 (53%)
	40 or over	11 (5%)	1765 (4%)
	Missing data	1 (0.1%)	0 (0%)

*Booking classifications are presented as defined by The National Institute for Health and Care Excellence (NICE)(22)

Whilst we set out to compare the ethnicity of women experiencing BBA with the overall South West data, this was prevented by the use of different ethnicity categories in the two data sets. For those experiencing BBA recorded ethnicity was: British European 94%, East European 2%, Other 2%, Indian 0.5%, North European 0.5%, South African 0.5%, South European 0.5%. For those in the South West data set ethnicity was: White 84%, Asian or Asian British 3%, Black or Black British 2%, Mixed 2%, Any other ethnic group 2%. We were also unable to consider social deprivation, as we had hoped to do, as we were only provided with the first half of patients' postcodes by the Trust. This was done to maintain anonymity of patient data.

Inequalities in neonatal hypothermia/poor outcomes

Hypothermia on arrival at hospital was more prevalent for pre-term babies, babies born to mothers who had not had a previous live birth, and babies born to mothers who reported a disability or safeguarding concern at booking (Table 3).

For peer review only

Table 3: Characteristics of mothers and babies, by neonatal temperature on arrival at hospital, n = 184

Characteristic of baby/mother		<36.5°C n = 64 Number (%)	≥36.5°C n = 120 Number (%)
Prematurity	Pre-term (<37 weeks)	9 (14%)	2 (2%)
	Term (37+ weeks)	54 (84%)	117 (98%)
	Unknown	1 (2%)	1 (1%)
Previous live birth	Yes	48 (75%)	109 (91%)
	No	16 (25%)	11 (9%)
Disability reported at booking	Yes	6 (9%)	2 (2%)
	No	58 (91%)	116 (96%)
	Unknown	0 (0%)	2 (2%)
Safeguarding concerns reported at booking	Yes	19 (30%)	20 (17%)
	No	45 (70%)	100 (83%)
Gestation at booking	Early booker (up to 12+6 weeks)	51 (80%)	102 (85%)
	Delayed booker (13 to 19+6 weeks)	6 (9%)	13 (11%)
	Late booker/unbooked (≥20 weeks)	6 (9%)	3 (2%)
	Unknown	1 (2%)	2 (2%)
Mother's ethnicity	British European	58 (91%)	114 (95%)
	East European	3 (4.5%)	0 (0%)
	Indian	0 (0%)	1 (1%)
	North European	1 (1.5%)	0 (0%)
	South African	1 (1.5%)	0 (0%)
	South European	0 (0%)	1 (1%)
	Other	1 (1.5%)	4 (3%)
Mother born in UK	Yes	57 (89%)	113 (94%)
	No	6 (9%)	5 (4%)
	Unknown	1 (2%)	2 (2%)
Model of care	Consultant-led	25 (39%)	48 (40%)
	Midwifery-led	37 (57%)	71 (59%)
	Unknown	2 (3%)	1 (1%)
Mother's attendance at antenatal appointments	All attended	52 (81%)	88 (73%)
	Not all attended	12 (19%)	26 (22%)
	Unknown	0 (0%)	6 (5%)
**Characteristic of baby/mother**		**<36.5°C**	**≥36.5°C**

For peer review only

		n = 64<br>Mean (SD)	n = 120<br>Mean (SD)
Mother's age at booking		28 (6.5)	31 (5.6)

Babies with neonatal hypothermia on arrival at hospital were more likely to be admitted to a Neonatal Unit (NNU) or receive advanced care (33% vs 19%). Advanced care was classified as any care needed in addition to 'normal newborn care', for example the provision of oxygen or intravenous (IV) medication.(23)

Of the hypothermic babies needing advanced care/admission to an NNU, a slight majority were born at term (11/21; 52%), one had a diagnosis of sepsis confirmed (5%), and just over half (11/21; 52%) were either moderately or severely hypothermic on arrival.

Seven (11%) hypothermic babies had an extended length of stay in hospital (>7 days) whereas none of the warm babies did. Due to the small sample size we were unable to perform in-depth statistical analysis to control for potential confounding variables. However, most (6/7, 8%) of the babies with an extended length of stay were also preterm. More treatment was given to hypothermic babies (glucose: 8% vs 1%; radiant heat source: 20% vs 3%; and incubator: 11% vs 3%).

Phase B: EMS call-handler advice on neonatal temperature management

Participant characteristics

Eighteen NHS staff took part in the four staff focus groups. Most participants were female (13/18, 72%). Participants' job titles comprised: midwives, paramedics, call-handlers, learning development officer, neonatal nurses, neonatal doctors, advanced care practitioners.

Twenty-two members of the public who had experience of prehospital birth took part in five focus groups. Most participants were female (18/22, 82%). Participants had a range of experiences, representing diverse geographical regions, areas of deprivation, and prehospital birth experience (e.g., at home, in the car, outside).

Five main themes were identified as affecting neonatal temperature management during EMS calls. These included: 1) the importance placed on neonatal temperature; 2) advice on where the baby should be placed following birth; 3) advice on how to keep the baby warm; 4) the timing of temperature management advice; and 5) the clarity and priority of instructions. Illustrative quotes are provided in Table 4.

Table 4: Illustrative quotes from qualitative data from focus groups

Quote no.	Focus Group	Quote
1	Public Focus Group 2	Nothing was mentioned about keeping the baby warm and to be honest, I had no idea that that's something that I should be doing.
2	Public Focus Group 2	When I delivered my daughter, their concern was mainly on delivering the placenta. It was my mum that had suggested that I should take my baby into my jumper that I was wearing to keep her warm because it was a cold morning. It was only until they realised that they couldn't deliver my placenta that they told me to get the blanket out and then wrap my daughter.
3	Staff Focus Group 2	You'll find that people want something to do. They want to focus in on something. And if you were to give that information around the importance of thermal regulation 'keeping that baby warm is really

For peer review only

		important now/ When that baby's born, we really want to keep that baby warm.' I think that would be really useful for that person who's trying to do something positive.
4	Staff Focus Group 2	Often when we're reviewing cases, we'll kind of note that as we kind of arrived on scene, the baby is on the floor and the mother's just in shock and the babies on a cold floor and it's drafty.
5	Public Focus Group 4	What we found really really confusing is that all the way through we were advised skin-to-skin, and don't do anything with the cord - keep that connected as long as possible. But then when it came to the actual 999 caller, it was totally different advice and it was 'keep the baby on the back'…Having that totally conflicting advice put my partner in the position where he was like 'I'm totally confused'.
6	Public Focus Group 4	When the baby did come out, she was just lying on the bed on her back. And I instinctively wanted to take her and put her on my chest like I did before with the midwives, and the instruction was to leave her on her back. I wasn't allowed to pick her up.
7	Public Focus Group 2	I was in a shopping mall, and there were lots of people around me. Lots of people looking and crowding. So I couldn't feel like I could do skin-to-skin. I was in panic and the security guy was a man. I'm sad I didn't get to do it, but it was too much stress.
8	Public Focus Group 4	I completely did not think straight in that moment at all…I was just a bit of an idiot, to be honest. And so when he came out, I didn't think about keeping him warm…I don't think you can rely on people thinking straight in that moment. … I think sometimes like I said, stating the obvious is quite useful in that kind of scenario.
9	Public Focus Group 4	We had quite a long wait before the midwife came. My husband was really panicking…but actually if someone had said to him 'move to a warm room' or 'go and put the heating on, if you haven't got it on already', he would have done that straight away.
10	Staff Focus Group 1	The gap should be used to paraphrase back to them. Just saying, you know…like 'dry, warm and wrapped'. Just keeping it simple repeating it back to them, showing that they understand the instruction for…however long it takes [the paramedics] to finally get there.
11	Public Focus Group 4	If it seems like it's a rushed labour, then perhaps maybe focus on what's going on at hand. And then perhaps maybe give the [temperature] advice thereafter like step by step... Whereas if it's maybe not as quick, maybe strike some sort of conversation with the person that's with the mother and say 'ok, when the baby does come out, remember to keep the baby warm.
12	Public Focus Group 1	If they can [provide warming advice] straight away if they've got time, then yeah, do it again also in between [gaps in the conversation]. If they've got time waiting for the ambulance, just mention it again. Just maybe check, check baby's chest or, you know, make sure it's warm or just little things like that because you do, you do forget.
13	Staff Focus Group 4	Babies being born in the sac is really rare. Usually it's you know, contractions, suddenly the water's gone, and the baby follows it quite quickly with these mums…So I don't think that should even be in there really because that might even worry mums like 'well, should there be a sac? Is it wrong that they're not in a sac? Oh my goodness me, you know, where's the sac?' And then start looking for a sac.

For peer review only - http://bmjopen.bmj.com/site/about/guidelines.xhtml

Theme 1: The importance placed on neonatal temperature

Although call-handlers from both triage systems repeated the instruction to 'keep mum and baby warm' at several points during the EMS call, participants noted that the importance of neonatal temperature was not explicitly addressed. This was of concern to participants who had experienced a BBA, who said that they were unaware of how quickly babies could become hypothermic in the prehospital setting, or how this could affect neonatal outcomes. It was noted by participants that antenatal information did not provide much information on neonatal hypothermia so they were unprepared for it (Q1).

Most participants reported that call-handlers had focused their advice and instructions on the person giving birth (e.g., determining whether the placenta had been delivered/whether there was any bleeding), and that limited attention had been given to the baby once it was established that they were breathing. Some reported that their baby had become hypothermic following their BBA and expressed frustration that it had not been made a priority or been communicated how important it was to keep their baby warm (Q2).

All participants felt that the importance of temperature could be further highlighted. Participants who had experienced prehospital birth suggested that this should be communicated in clear and simple language. NHS staff suggested that giving the caller the 'job' of keeping the baby warm could help emphasise this importance and increase the chance of temperature management being performed well (Q3).

Theme 2: Where the baby should be placed following birth

The two triage systems differed in their instruction on where the baby should be placed following the birth. The AMPDS triage system states that callers should 'keep the baby between the mother's legs and level with her bottom', while NHS Pathways states that callers should 'place the baby on the mother's chest or tummy'.

Participants expressed frustration at the instruction to place the baby between the mother's legs and felt this could be very detrimental to temperature management, especially if the mother was in a room with a cold floor, such as a bathroom or kitchen (Q4).

Participants who had received this advice following prehospital birth said that it had caused confusion, as it contradicted what they had been taught antenatally (i.e., skin-to-skin contact following birth) (Q5).

For those who had given birth in a car, this advice had proven especially challenging. One participant noted that by trying to keep the baby between her legs her husband had had to open all the car doors, which had potentially contributed to her baby being hypothermic on arrival at hospital.

Some participants spoke of not being 'allowed' to pick their baby up following the BBA and to keep their baby on its back on the bed/floor until the paramedics arrived (Q6).

Skin-to-skin contact with the mother was put forward by NHS staff as the optimal practice for neonatal normothermia following prehospital birth. There was some discussion amongst staff regarding the importance of maintaining a clear airway during skin-to-skin contact, and it was acknowledged that regular prompts would need to be given by call-handlers to check that the baby's nose and mouth could be seen. Participants also emphasised the need for clear and simple instructions to be given by call-handlers on how to conduct skin-to-skin contact effectively, including checking to see if the mother was warm themselves.

For peer review only - http://bmjopen.bmj.com/site/about/guidelines.xhtml

Most participants said that skin-to-skin contact would have been practical in the circumstances surrounding their BBA. Some circumstances were seen as potentially problematic for skin-to-skin contact, for example, if the cord was too short, they were too anxious, or were not confident in handling the baby (e.g., first-time parents). Some participants expressed concerns over privacy regarding skin-to-skin contact and said that they would not feel confident doing this in a public place or if they thought male paramedics might attend (Q7).

Staff also acknowledged that there may be cases where skin-to-skin contact was not appropriate or possible, for which staff suggested thorough drying and wrapping of the baby before placing them on someone warm.

Ensuring that the baby was put in the optimal place was discussed by participants as particularly important in the context of long wait times for ambulances and the “cost of living crisis”. Participants noted that people could potentially be waiting for hours for an ambulance in a cold house, so ensuring the baby is kept as warm as possible should be a priority when advising callers where to place the baby following birth.

Theme 3: How to keep the baby warm
Neither of the two triage systems contain specific advice on how to keep the baby warm, although some call-handlers did add some advice themselves during calls (e.g., suggesting the use of a hat). Generic advice of ‘keep mum and baby warm’ was given by each pathway, and callers were also told to ‘cover the baby’s head, but not its face’ and to ‘use towels and blankets to keep the baby warm’.

During focus groups with NHS staff, participants expressed a preference for warming advice to be more specific and targeted. Suggestions included advising skin-to-skin contact (as above), putting a hat on the baby, considering possible changes to the environment (e.g., closing any windows, moving off cold floors, putting the heating on), and emphasising the need to dry the baby thoroughly and replace wet towels with dry ones. Participants also suggested adding an instruction for callers to check the warmth of the baby by touching the skin on the chest/back, to see if warming measures were working.

The specific advice put forward by staff was viewed positively in focus groups with those who had experienced a BBA. Many suggested that they had experienced stress and anxiety during their BBA, and that this had caused them to ‘lose all common sense’ or be unable to think rationally. As such, the instruction to ‘keep the baby warm’ was viewed as too generic and ambiguous by participants, who felt that clear, practical instructions would be easier to follow and would help them to feel more in control of the situation (Q8).

Most participants felt that all warming measures suggested by staff would have been practical in the circumstances of their BBA, and that alternatives would have been easily considered if not (e.g., using an item of clothing instead of a blanket/towel). Participants did acknowledge, however, that some warming measures may not be practical in all situations, and that callers should be advised that each warming measure should be carried out ‘if possible’. Participants also noted that listing all warming measures may not be appropriate if the woman was calling for herself and had no-one around to assist her.

Of the measures suggested by staff, potential changes to the environment were emphasised by participants as something that could have considerably influenced their baby’s temperature. Participants suggested that simple advice such as moving to a warmer room would have given them

For peer review only - http://bmjopen.bmj.com/site/about/guidelines.xhtml

a sense that they were positively impacting the situation and helped relieve some of the anxiety and panic (Q9).

Theme 4: Timing of temperature management advice

Another key theme about the content and structure of the EMS triage systems was the timing of neonatal temperature management advice. While call-handlers from both pathways focused this advice predominantly after the baby was born, long gaps were identified in some calls while call-handlers waited for a part of the baby to be seen by the caller. NHS staff suggested that these gaps could be used to emphasise the importance of temperature, and to give advice on warming the environment if appropriate. Gaps in the conversation were also identified during periods of time where call-handlers had completed the triage algorithm and were waiting for paramedics to arrive. These gaps were often filled with small-talk. Staff suggested that this would be an appropriate time to give specific warming advice, and for call-handlers to check that essential warming measures had been completed (Q10).

Participants who had experienced a BBA felt that warming advice should be given as soon as possible, but without interrupting vital instructions. Some participants felt that the woman should be the priority and focus of calls before the baby was born, but that a brief mention of the importance of temperature would be helpful, and that focus should then shift to keeping the baby warm after birth (Q11).

Participants agreed with NHS staff that once the triage algorithm was complete, any additional time spent on calls waiting for the ambulance should be used to provide specific warming advice and to check it had been followed appropriately. Participants felt that it would be helpful to be reminded of all the steps they could take to keep their baby warm at any possible opportunity (Q12).

Focus group participants also discussed advice that could be given antenatally to prepare them for neonatal temperature management in the prehospital setting. Suggestions included an antenatal leaflet on the importance of neonatal thermoregulation, a reminder at antenatal appointments, and/or advice to pack a blanket in their hospital bag (in case of birthing in the car).

Theme 5: Clarity and priority of instructions

The clarity and priority given to instructions was discussed by participants in both focus groups. Several instructions were highlighted by participants as potentially confusing or distracting, and some felt that the timing and priority given to these instructions could delay neonatal temperature management. For example, calls from the NHS Pathways algorithm included instructions for the caller to 'wipe or peel away the sac' before drying and wrapping the baby. This frequently caused confusion to callers, as none of the babies were born in their sac and meant that drying and wrapping instructions were delayed while the call-handler and caller discussed the nature of the sac. NHS staff suggested that the previous instruction to 'wipe the baby's nose and mouth' should be sufficient to cover any issues with the sac and suggested that this instruction be removed or altered to avoid confusion. Participants from both sets of focus groups suggested that once the baby was born, only essential instructions should be given before warming advice, as to avoid any confusion or distraction (Q13).

Other instructions questioned by NHS staff included the advice to get towels and blankets to 'protect the bed or floor', and the caller being directed to wash their hands once a part of the baby could be seen.

DISCUSSION

For peer review only

This is the first study, to our knowledge, to investigate the temperatures of babies arriving at hospital following a prehospital birth (birth before arrival; BBA) in South West England. We found that 35% of babies born prehospital arrived at hospital hypothermic (<36.5°C), and 25% of these were classed as 'moderately' or 'severely' hypothermic, with warming needed urgently to prevent poor outcomes. These findings are in line with those of a study in Finland, which found hospital diagnosis of neonatal hypothermia to be recorded in 53% of babies born out of hospital, compared to only 0.7% of babies born in hospital.(4) Our findings also concur with research from France, which found that 56% of babies born outside of hospital were hypothermic on arrival at hospital,(16) and that neonatal hypothermia was associated with increased rates of NICU admission.(5) Although our findings suggest better rates of neonatal normothermia following BBA than literature from other areas, 35% remains a significant proportion of babies arriving at hospital hypothermic. This could potentially be improved through better availability of optimal equipment, further training, increased awareness amongst ambulance staff,(10) and improvements in EMS call-handler advice.

We found that particular babies may be more at risk of hypothermia on hospital arrival following BBA. These include pre-term babies, babies of first-time mums, babies of women with safeguarding concerns, and babies of women who reported disabilities at their booking appointment. While prematurity and low birthweight are established risk factors for hypothermia in newborns generally,(24,25) there is a paucity of previous literature exploring the other potential risk factors we identified. While our study is limited by the small sample size and focused geographical location. these preliminary associations provide a basis for future research, and may help NHS Trusts identify babies who require additional warming and support following a BBA. Further work should replicate and expand our study to consider areas of high population density, with a larger sample size to enable a more detailed statistical analysis.

Our findings suggest that there may be some women in South West England who are more likely to experience BBA, which previous research suggests may result in poorer outcomes for their babies.(4,5) These include women with safeguarding concerns, women who book their pregnancies with a health professional later than 13 weeks, and those who have had babies before. Multiparity, late booking and/or lack of antenatal care are known to be associated with BBA from previous literature,(2,4,12,26) however safeguarding concerns at booking has not been identified previously. Due to the small sample size of this study, caution should be taken when interpreting results but this finding may help maternity services identify and support women who are more likely to experience BBA. Despite receiving information on mothers' ethnicity from our BBA data, we were unable to explore any associations, as local ethnicity data were recorded differently to the National Maternity Data Set.(21) Work is ongoing to resolve this issue.

This study is the first to investigate neonatal temperature management advice provided by EMS call-handling triage systems. EMS dispatching is complex, and a call-handler must triage the call to inform the prioritisation of resource dispatch whilst also providing first aid advice prior to the arrival of an EMS resource (pre-arrival advice).(27) Our research identifies issues with unclear and contradictory pre-arrival advice during UK EMS calls regarding BBA, and suggests five themes that could influence neonatal thermal care during these calls. NHS staff and members of the public made several recommendations for changes to the script to address potential barriers to good thermal care, based on these themes. These changes have already been implemented in NHS Pathways, and similar changes are pending approval in AMPDS.

Although this is the first study to investigate neonatal temperature management advice given by EMS call-handlers, increasing attention is being paid to call-handler advice and interactions with patients,(28) especially during maternity cases.(17) Indeed, the Healthcare Safety Investigation

For peer review only - http://bmjopen.bmj.com/site/about/guidelines.xhtml

Branch (HSIB) report into 'Maternity pre-arrival instructions by 999 call handlers'(17) similarly found that the two triage systems in England provide different pre-arrival instructions for birth, associated with different implications and risk. There is a growing body of evidence investigating the call-handler and caller interaction during EMS calls,(28) and this research should be considered in any proposed changes to emergency maternity pre-arrival advice. While much of the research into call-handler and caller interaction during the EMS call has focused on the out-of-hospital cardiac arrest (OHCA) scenario, these findings can be used to inform emergency maternity pre-arrival instructions, and align with our own findings. For example, simplified instructions have been associated with a shorter interval between instruction and first chest compression in telephone cardiopulmonary resuscitation instruction in OHCA.(29) Future research could usefully investigate the design, implementation, and impact of emergency maternity pre-arrival advice.

CONCLUSION

The findings of this study suggest there may be certain women who are more likely to have a BBA in South West England and certain babies who are more likely to be hypothermic on arrival at hospital following a BBA. Findings also suggest potential areas for improvement to EMS call-handler advice regarding neonatal temperature management. Future work in this area has the potential to reduce the number of babies arriving at hospital hypothermic and improve longer term outcomes, thereby addressing health inequalities by disproportionately benefitting underserved groups.

LEGENDS

Figure 1: Flowchart to show the process of how call-handler data and focus group data were identified, screened, and analysed.

STATEMENTS

Acknowledgments

We would like to thank the NHS staff and members of the public who took part in focus groups for this evaluation, as well as the NHS Hospital and Ambulance Service Trusts who provided data.

We would also like to thank: Alice Jennings from UWE Bristol, for supporting the collection of hospital data and contributing to analysis of EMS calls; Sally Hedge from the South West AHSN for supporting and promoting this work; and the members of our Patient and Public Involvement Group for their invaluable input and contributions to this study: Tasha Cooper, Farzana Kausir, Poonam Barnes, Louise Walker, Simon Walker and Arif Hoque.

Finally, we would like to thank the funder of this project, the South West AHSN, and the study sponsor, UWE Bristol. This work was carried out by members of the Research in Emergency Care, Avon Collaborative Hub (REACH Bristol).

Patient and Public Involvement

Patients and/or the public were involved in the design, or conduct, or reporting, or dissemination plans of this research. A dedicated PPI group guided this work from its inception, supporting the development of research questions and study design. The group consisted of four women and two men who had experience of BBA. Experiences of the team were varied and represented ethnic,

For peer review only

cultural and geographical diversity. Over the course of the project, the PPI group reviewed and refined participant facing documents for stakeholder focus groups, contributed to data synthesis, and advised the team on the approach to recruitment and dissemination. They also read and commented on the draft of this manuscript. Findings were shared with both the PPI group and study participants via email, presentations, and national conferences.

Patient consent for publication
Not required

Ethics committee approval
Ethics committee approval for this service evaluation was granted by the University of the West of England (UWE Bristol) Faculty of Health and Applied Sciences Research Ethics Committee (HAS.22.01.061). Participants gave informed consent to participate in the study before taking part.

Provenance and peer review
Not commissioned; externally peer reviewed.

Contributorship statement
LG was the Chief Investigator/guarantor with overall responsibility for the study, and participated in study conception, design and coordination, performed and coded the focus groups, analysed the data and drafted the manuscript. HT participated in study conception, design and interpretation of the results, and supported quantitative data analysis. GM, KK, EB, AB, TD, RO, HM, RMC, NM, SV and JB participated in study conception, design and interpretation of the results. All authors were responsible for the critical revision of the manuscript for publication and approved the final version to be published.

Competing interests
None declared

Funding
The study was funded by the South West Academic Health Science Network (AHSN) through its Perinatal Equity programme.

Data sharing statement
Data are available upon reasonable request.

REFERENCES

1. Office for National Statistics. *Vital Statistics in the UK: Births, Deaths and Marriages* [online]. 2019. https://www.ons.gov.uk/peoplepopulationandcommunity/populationandmigration/populationestimates/datasets/vitalstatisticspopulationandhealthreferencetables (accessed January 17, 2024).

2. Loughney A, Collis R, Dastgir S. Birth before arrival at delivery suite: associations and consequences. *Br J Midwifery*. 2006;14(4):204-208.

3. Snowden JM, Tilden EL, Snyder J, et al. Planned out-of-hospital birth and birth outcomes. *New England Journal of Medicine*. 2015;373(27):2642-2653.

For peer review only - http://bmjopen.bmj.com/site/about/guidelines.xhtml

4. Ovaskainen K, Ojala R, Gissler M, et al. Out-of-hospital deliveries have risen involving greater neonatal morbidity: risk factors in out-of-hospital deliveries in one University Hospital region in Finland. *Acta Paediatr*. 2015;104(12):1248-1252.

5. Javaudin F, Hamel V, Legrand A, et al. Unplanned out-of-hospital birth and risk factors of adverse perinatal outcome: findings from a prospective cohort. *Scand J Trauma Resusc Emerg Med*. 2019;27(1):26.

6. Nguyen ML, Lefevre P, Dreyfus M. Maternal and neonatal outcomes of unplanned deliveries. *J Gynecol Obstet Biol Reprod (Paris)*. 2016;45(1):86-91. doi:10.1016/j.jgyn.2015.02.002

7. Chitty H, Wyllie J. Importance of maintaining the newly born temperature in the normal range from delivery to admission. In: *Seminars in Fetal and Neonatal Medicine*. Vol 18. Elsevier; 2013:362-368.

8. World Health Organization. *Thermal Control of the Newborn: A Practical Guide* [online]. 1993. https://apps.who.int/iris/bitstream/handle/10665/60042/WHO_FHE_MSM_93.2.pdf (accessed March 13, 2023).

9. Perlman JM, Wyllie J, Kattwinkel J, et al. Part 7: neonatal resuscitation: 2015 international consensus on cardiopulmonary resuscitation and emergency cardiovascular care science with treatment recommendations. *Circulation*. 2015;132(16,1):S204-S241.

10. Goodwin L, Voss S, McClelland G, et al. Temperature measurement of babies born in the pre-hospital setting: analysis of ambulance service data and qualitative interviews with paramedics. *Emergency Medicine Journal*. 2022;39(11):826-832.

11. McClelland G, Burrow E, McAdam H. Babies born in the pre-hospital setting attended by ambulance clinicians in the north east of England. *Br Paramed J*. 2019;4(3):43-48.

12. Renesme L, Garlantézec R, Anouilh F, et al. Accidental out-of-hospital deliveries: a case–control study. *Acta Paediatr*. 2013;102(4):174-177.

13. Diana L, Glaucia L, Adriana C, et al. Out-of-hospital deliveries: A case-control study. *Turkish Archives of Pediatrics/Türk Pediatri Arşivi*. 2018;53(2):87.

14. Moscovitz HC, Magriples U, Keissling M, et al. Care and Outcome of Out-of-hospital Deliveries. *Academic Emergency Medicine*. 2000;7(7):757-761.

15. Adamsons K, Towell ME. Thermal homeostasis in the fetus and newborn. *The Journal of the American Society of Anesthesiologists*. 1965;26(4):531-548.

16. Javaudin F, Roche M, Trutt L, et al. Assessment of rewarming methods in unplanned out-of-hospital births from a prospective cohort. *Scand J Trauma Resusc Emerg Med*. 2020;28:1-9.

17. Healthcare Safety Investigation Branch. *Maternity Pre-Arrival Instructions by 999 Call Handlers. Independent Report by the Healthcare Safety Investigation Branch NI-000818* [online]. 2022. https://hsib-kqcco125-media.s3.amazonaws.com/assets/documents/hsib-report-maternity-pre-arrival-instructions-by-999-call-handlers.pdf (accessed August 21, 2023).

18. Health Innovation South West. *Health Innovation South West* [online]. 2022. https://healthinnovationsouthwest.com (accessed September 21, 2023).

For peer review only - http://bmjopen.bmj.com/site/about/guidelines.xhtml

19. Patton MQ. *Qualitative Research & Evaluation Methods: Integrating Theory and Practice*. Sage publications; 2014.

20. Malterud K, Siersma VD, Guassora AD. Sample size in qualitative interview studies: guided by information power. *Qual Health Res*. 2016;26(13):1753-1760.

21. NHS Digital. *NHS Maternity Statistics, 2021-2022: MSDS CSV Data* [online]. 2022. https://digital.nhs.uk/data-and-information/publications/statistical/nhs-maternity-statistics/2021-22 (accessed March 13, 2023).

22. National Collaborating Centre for Women's and Children's Health. *NICE Clinical Guideline: Pregnancy and complex social factors: a model for service provision for pregnant women with complex social factors* [online]. 2010. https://www.nice.org.uk/Guidance/CG110 (accessed May 20, 2023).

23. British Association of Perinatal Medicine. *A Framework for Neonatal Transitional Care*. 2017. https://www.bapm.org/resources/24-neonatal-transitional-care-a-framework-for-practice-2017 (accessed January 17, 2023).

24. Miller SS, Lee HC, Gould JB. Hypothermia in very low birth weight infants: distribution, risk factors and outcomes. *Journal of Perinatology*. 2011;31(1):49-56. doi:10.1038/jp.2010.177

25. Laptook AR, Salhab W, Bhaskar B, et al. Admission temperature of low birth weight infants: predictors and associated morbidities. *Pediatrics*. 2007;119(3):643-649.

26. Bhoopalam S, Watkinson M. Babies born before arrival at hospital. *BJOG*. 1991;98(1):57-64.

27. Møller TP, Jensen HG, Viereck S, Lippert F, Østergaaard D. Medical dispatchers' perception of the interaction with the caller during emergency calls-a qualitative study. *Scand J Trauma Resusc Emerg Med*. 2021;29(1):1-10.

28. Perera N, Finn J, Bray J. Can emergency dispatch communication research go deeper? *Resusc Plus*. 2022;9.

29. Leong PWK, Leong BSH, Arulanandam S, et al. Simplified instructional phrasing in dispatcher-assisted cardiopulmonary resuscitation–when 'less is more.' *Singapore Med J*. 2021;62(12):647.

For peer review only

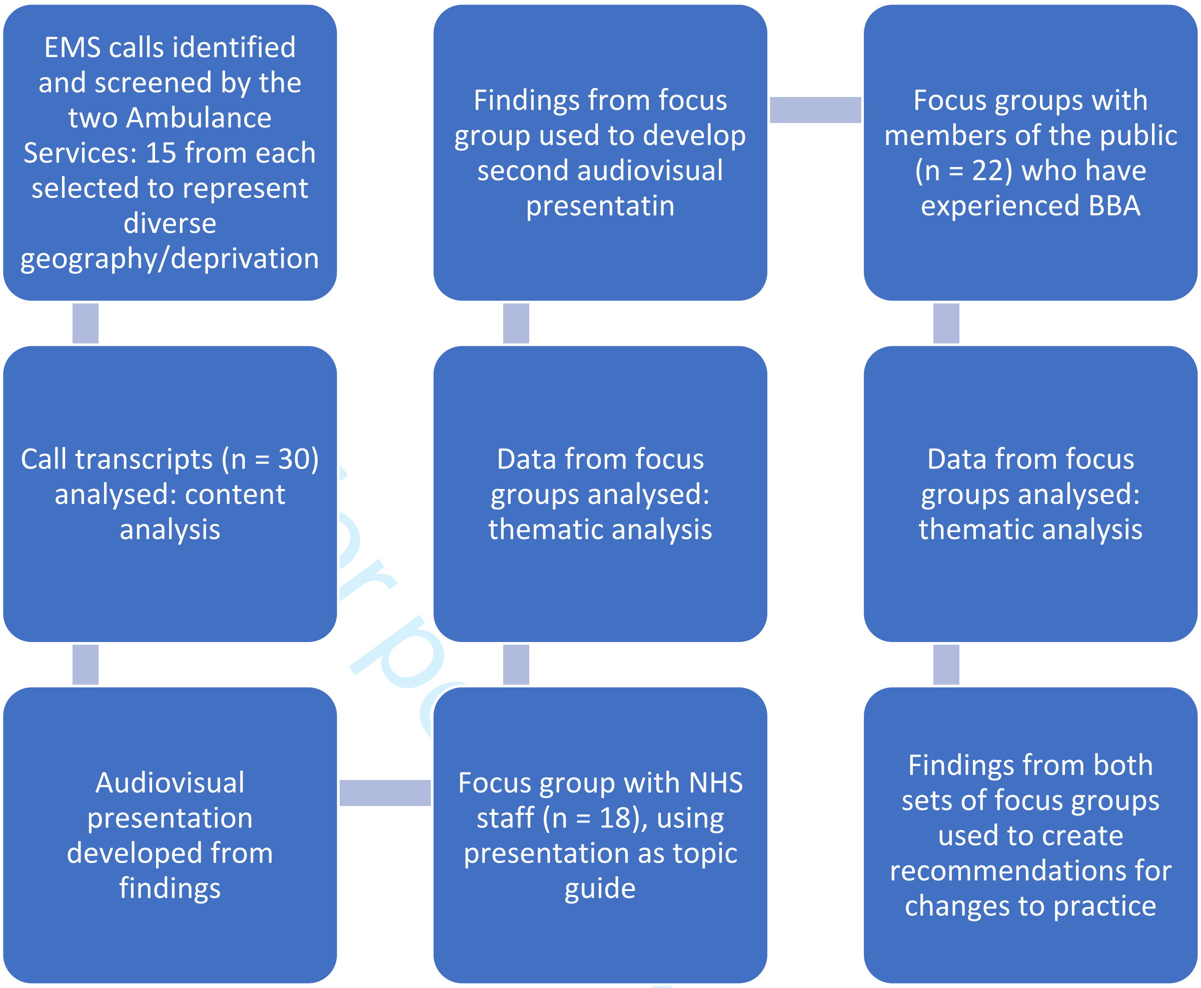

Figure 1: Flowchart to show the process of how call-handler data and focus group data were identified, screened, and analysed.

Supplemental File 1

Definitions of characteristics of mothers and babies

- Prematurity: recorded gestation at birth (weeks and days. Pre-term (<37 weeks); Term (37+ weeks).
- Previous live birth: self-reported by the mother and identified by the midwife.
- Disability reported at booking: any disability self-reported by the mother at the booking appointment.
- Safeguarding concerns reported at booking: any safeguarding concerns self-reported by the mother or identified by the midwife at the booking appointment.
- Gestation at booking: gestational age (weeks and days) recorded at the booking appointment.
- Mother's ethnicity: self-reported by the mother at the booking appointment.
- Mother born in UK: self-reported by the mother at the booking appointment.
- Model of care: recorded by the midwife.
- Mother's attendance at antenatal appointments: recorded by the midwife.
- Mother's age at booking: recorded by the midwife.

For peer review only

Supplementary Table 1: COREQ checklist

**Domain 1: Research team and reflexivity**		**Location in manuscript (Section, page no.)**
**Personal Characteristics**		
1. Interviewer/facilitator Which author/s conducted the interview or focus group?	LG	Methods - 5
2. Credentials<br>What were the researcher's credentials? E.g. PhD, MD	BSc, PhD	Title Page
3. Occupation<br>What was their occupation at the time of the study?	Senior Research Fellow in Emergency Care	Title Page
4. Gender Was the researcher male or female?	Female	N/A
5. Experience and training<br>What experience or training did the researcher have?	Experienced qualitative researcher.	Methods - 5
**Relationship with participants**		
6. Relationship established<br>Was a relationship established prior to study commencement?	No	N/A
7. Participant knowledge of the interviewer<br>What did the participants know about the researcher? e.g. personal goals, reasons for doing the research	Participants were briefed on the purpose of the study and were aware that ethical approval had been granted. Participants reviewed the participant information documentation prior to giving their written informed consent to be involved.	Methods - 5
8. Interviewer characteristics What characteristics were reported about the interviewer/facilitator? e.g. Bias, assumptions, reasons and interests in the research topic	No interviewer-related biases identified.	N/A
**Domain 2: study design**		
**Theoretical framework**		
9. Methodological orientation and Theory<br>What methodological orientation was stated to underpin the study? e.g. grounded theory, discourse analysis, ethnography, phenomenology, content analysis	Thematic analysis	Methods – 5
Participant selection		
10. Sampling How were participants selected? e.g.	Purposive sampling was used: recruitment adverts were circulated using social media and collaborating organisations	Methods - 5

11. Method of approach How were participants approached? e.g. face-to-face, telephone, mail, email	As above	Methods - 5
12. Sample size How many participants were in the study?	18 NHS staff and 22 members of the public	Results - 7
13. Non-participation How many people refused to participate or dropped out? Reasons?	There were no participants who subsequently refused to participate, withdrew consent or dropped out.	N/A
**Setting**		
14. Setting of data collection Where was the data collected? e.g. home, clinic, workplace	Data was collected and recorded via a Microsoft Teams meeting.	Methods - 5
15. Presence of non-participants Was anyone else present besides the participants and researchers?	No	N/A
16. Description of sample What are the important characteristics of the sample? e.g. demographic data, date	Most participants were female (72% of the NHS staff and 82% of the patients). For NHS staff, participants' job titles comprised: midwives, paramedics, call-handlers, learning development officer, neonatal nurses, neonatal doctors, advance care practitioners. For members of the public, participants had a range of experiences, representing diverse geographical regions, areas of deprivation, and prehospital birth experience (e.g., at home, in the car, outside).	Results – 7
**Data collection**		

For peer review only

17. Interview guide Were questions, prompts, guides provided by the authors? Was it pilot tested?	Focus group discussions were semi-structured using a PowerPoint presentation to guide discussion; the presentations were reviewed by the study team and Patient and Public Involvement group before focus groups took part.	Methods - 5
18. Repeat interviews Were repeat interviews carried out? If yes, how many?	No	N/A
19. Audio/visual recording Did the research use audio or visual recording to collect the data?	Focus groups were recorded Microsoft Teams.	Methods - 5
20. Field notes Were field notes made during and/or after the interview or focus group?	No additional field notes were made.	N/A
21. Duration What was the duration of the interviews or focus group?	The focus groups lasted an average of 60 minutes.	N/A
22. Data saturation Was data saturation discussed?	We used information power	Methods - 5
23. Transcripts returned Were transcripts returned to participants for comment and/or correction?	No	N/A
**Domain 3: analysis and findings**		
**Data analysis**		
24. Number of data coders How many data coders coded the data?	One - LG	Methods - 5
25. Description of the coding tree Did authors provide a description of the coding tree?	N/A – not used	N/A
26. Derivation of themes Were themes identified in advance or derived from the data?	Themes were derived from the data	Methods - 5
27. Software What software, if applicable, was used to manage the data?	N/A	N/A
28. Participant checking Did participants provide feedback on the findings?	No	N/A
**Reporting**		
29. Quotations presented Were participant quotations presented to illustrate the themes / findings? Was each quotation identified? e.g. participant number	Yes	Results – 7-11

For peer review only

30. Data and findings consistent<br>Was there consistency between the data presented and the findings?	Yes	N/A
31. Clarity of major themes<br>Were major themes clearly presented in the findings?	Yes	Results 7-11
32. Clarity of minor themes<br>Is there a description of diverse cases or discussion of minor themes?	Yes	Results 7-11

For peer review only

BMJ Open

Inequalities in birth before arrival at hospital in South West England: a multi-methods study of neonatal hypothermia and Emergency Medical Services call-handler advice

Journal:	*BMJ Open*
Manuscript ID	bmjopen-2023-081106.R2
Article Type:	Original research
Date Submitted by the Author:	15-Mar-2024
Complete List of Authors:	Goodwin, Laura; University of the West of England, School of Health and Social Wellbeing<br>Kirby, Kim; South Western Ambulance Service NHS Foundation Trust, Research, Audit and Quality Improvement Department; University of the West of England, School of Health and Social Wellbeing<br>McClelland, Graham; Northumbria University<br>Beach, Emily; University of the West of England, School of Health and Social Wellbeing<br>Bedson, Adam; South Western Ambulance Service NHS Foundation Trust<br>Benger, Jonathan; University of the West of England, School of Health and Social Wellbeing<br>Deave, Toity; University of the West of England, Centre for Child and Adolescent Health<br>Osborne, Ria; South Western Ambulance Service NHS Foundation Trust, Research, Audit & Improvement<br>McAdam, Helen; University of Sunderland, Health Sciences and Wellbeing<br>McKeon-Carter, Roisin; University Hospitals Plymouth NHS Trust<br>Miller, Nick; University of the West of England, School of Health and Social Wellbeing<br>Taylor, Hazel; University Hospitals Bristol NHS Foundation Trust, Research Design Service<br>Voss, Sarah; University of the West of England, School of Health and Social Wellbeing
**Primary Subject Heading**:	Emergency medicine
Secondary Subject Heading:	Obstetrics and gynaecology, Health services research, Paediatrics
Keywords:	ACCIDENT & EMERGENCY MEDICINE, Health Equity, NEONATOLOGY, Maternal medicine < OBSTETRICS, Paediatric A&E and ambulatory care < PAEDIATRICS

SCHOLARONE™
Manuscripts

I, the Submitting Author has the right to grant and does grant on behalf of all authors of the Work (as defined in the below author licence), an exclusive licence and/or a non-exclusive licence for contributions from authors who are: i) UK Crown employees; ii) where BMJ has agreed a CC-BY licence shall apply, and/or iii) in accordance with the terms applicable for US Federal Government officers or employees acting as part of their official duties; on a worldwide, perpetual, irrevocable, royalty-free basis to BMJ Publishing Group Ltd ("BMJ") its licensees and where the relevant Journal is co-owned by BMJ to the co-owners of the Journal, to publish the Work in this journal and any other BMJ products and to exploit all rights, as set out in our licence.

The Submitting Author accepts and understands that any supply made under these terms is made by BMJ to the Submitting Author unless you are acting as an employee on behalf of your employer or a postgraduate student of an affiliated institution which is paying any applicable article publishing charge ("APC") for Open Access articles. Where the Submitting Author wishes to make the Work available on an Open Access basis (and intends to pay the relevant APC), the terms of reuse of such Open Access shall be governed by a Creative Commons licence – details of these licences and which Creative Commons licence will apply to this Work are set out in our licence referred to above.

Other than as permitted in any relevant BMJ Author's Self Archiving Policies, I confirm this Work has not been accepted for publication elsewhere, is not being considered for publication elsewhere and does not duplicate material already published. I confirm all authors consent to publication of this Work and authorise the granting of this licence.

For peer review only

Inequalities in birth before arrival at hospital in South West England: a multi-methods study of neonatal hypothermia and Emergency Medical Services call-handler advice

For peer review only

Authors:

Goodwin, L.[1], Kirby, K.[1,2], McClelland, G.[3], Beach, E.[1], Bedson, A.[3], Benger, J.[1], Deave, T.[1], Osborne, R.[3], McAdam, H.[4], McKeon-Carter, R.[5], Miller, N.[1], Taylor, H.[6], & Voss, S.[1]

Institutions:

[1] University of the West of England, Bristol
[2] South Western Ambulance Service NHS Foundation Trust
[3] Northumbria University
[4] University of Sunderland
[5] University Hospitals Plymouth NHS Trust
[6] University Hospitals Bristol and Weston NHS Foundation Trust

Corresponding author:
Kim Kirby – Kim.Kirby@uwe.ac.uk
Keywords: Infant, Newborn, Ambulances, Hypothermia, Temperature, Emergency Service, Hospital

List of abbreviations:

AHSN – Academic Health Science Network
BBA – Birth Before Arrival at hospital
PPI – Patient and Public Involvement
SWASFT – South Western Ambulance Service NHS Foundation Trust
UK – United Kingdom

Word count: 5284

For peer review only - http://bmjopen.bmj.com/site/about/guidelines.xhtml

ABSTRACT

Objectives: To examine inequalities in birth before arrival (BBA) at hospitals in South West England, understand which groups are most likely to experience BBA and how this relates to hypothermia and outcomes (Phase A). To investigate opportunities to improve temperature management advice given by Emergency Medical Services (EMS) call-handlers during emergency calls regarding BBA in the United Kingdom (Phase B).

Design: A two-phase multi-method study. Phase A analysed anonymised data from hospital neonatal records between January 2018 and January 2021. Phase B analysed anonymised EMS call transcripts, followed by focus groups with National Health Service (NHS) staff and patients.

Setting: Six Hospital Trusts in South West England, and two EMS providers (ambulance services) in South West and North East England.

Participants: Eighteen multidisciplinary NHS staff and 22 members of the public who had experienced BBA in the UK.

Results: 35% (64/184) of babies conveyed to hospital were hypothermic on arrival. When compared with national data on all births in the South West, we found higher percentages of women with documented safeguarding concerns at booking, previous live births, and 'late bookers' (booking their pregnancy >13 weeks gestation). These women may therefore be more likely to experience BBA. Pre-term babies, babies to first-time mothers, and babies born to mothers with disability or safeguarding concerns at booking were more likely to be hypothermic following BBA. Five main themes emerged from qualitative data on call-handler advice: 1) importance placed on neonatal temperature; 2) advice on where the baby should be placed following birth; 3) advice on how to keep the baby warm; 4) timing of temperature management advice; 5) clarity and priority of instructions.

Conclusions: Findings identified factors associated with BBA and neonatal hypothermia following BBA. Improvements to EMS call-handler advice could reduce the number of babies arriving at hospital hypothermic.

STRENGTHS AND LIMITATIONS OF THIS STUDY

- To the best of our knowledge, this is the first study to investigate the temperature of babies arriving at hospital following a prehospital birth in South West England.
- This is the first study to investigate neonatal temperature management advice provided by emergency service call-handling triage systems.
- The service evaluation of hospital neonatal records had a small sample size and was confined to one geographical area which limits the representativeness of our findings.
- Focus groups represent the views of a small sample of participants and this may limit the transferability of the findings
- Our research was conducted using data that spanned the COVID-19 pandemic when UK maternity services were significantly impacted and this may have influenced our findings

FUNDING STATEMENT

The study was funded by the South West Academic Health Science Network as part of its Perinatal Health Equity programme.

COMPETING INTERESTS

None declared

For peer review only

BACKGROUND

In the UK, an estimated 3,700 births a year are unplanned out of hospital births, or Births Before Arrival at hospital (BBA).[1,2] These births are attended by ambulance staff such as paramedics, emergency care assistants and ambulance technicians, usually without the attendance of a qualified midwife or obstetrician. BBA is associated with unfavourable perinatal outcomes and increased mortality.[3-5]

An important risk factor for mortality following BBA is hypothermia,[5-7]which is defined by the World Health Organization (WHO) as a temperature below 36.5°C.[8] The mortality risk from hypothermia is especially prominent in premature babies; in low birth weight infants, mortality increases by 28% per 1°C decrease in admission temperature below 36.5°C.[9] Paramedics are usually the first on scene at BBAs, however recent audits of Emergency Medical Service (EMS) data in the UK suggest that paramedics do not routinely take newborn temperatures (temperatures recorded in 2-10% of cases).[10,11] When temperatures have been recorded, the majority have indicated neonatal hypothermia (72%).[10,11] Currently there are no published UK data to show the percentage of babies who are hypothermic on arrival to hospital following a BBA, and the resulting outcomes for these babies.

To prevent neonatal hypothermia and poor outcomes associated with neonatal hypothermia following BBA, it is important to understand which women are most likely to experience BBA, which babies are most likely to be cold on arrival, and how this interacts with outcomes such as mortality, length of hospital stay, and the need for special care and interventions. Literature around inequalities in maternal and perinatal outcomes suggests an increased risk of poor outcomes for women who do not engage or have little engagement with maternity services.[2,12] This includes concealed or teenage pregnancies, as well as women from migrant or minority ethnic backgrounds.[13,14] A call to EMS regarding labour or birth may, therefore, be the first point of contact that some women will have regarding their pregnancy.

In the prehospital setting, babies can become hypothermic within minutes[15] and are sometimes born before EMS arrival. In these instances, the newborn is more likely to be hypothermic on arrival at hospital, potentially due to ineffective warming by non-professionals.[16] Therefore, the earlier that effective warming measures can be initiated (for example at the point of calls to EMS), the better the chances of improved outcomes following BBA. A recent Healthcare Safety Investigation Branch (HSIB) report[17] highlighted the fact that UK investigations into poor patient outcomes following BBA do not routinely consider the clinical impact of advice given by EMS call-handlers or recommended work with relevant stakeholders to develop guidance for maternity emergencies during these calls. It is necessary to investigate the advice given regarding neonatal temperature management during EMS calls relating to BBA, and work with relevant stakeholders to investigate potential ways to develop and improve this advice.[17]

The aims of this project were:

1) To examine inequalities in BBA in South West England and understand which groups are most likely to experience BBA, and also how this relates to hypothermia and neonatal outcomes (Phase A).
2) To investigate opportunities to improve the temperature management advice given by EMS call-handlers during emergency calls regarding BBA in the UK (Phase B).

For peer review only - http://bmjopen.bmj.com/site/about/guidelines.xhtml

METHODS

Setting

Hospital data were collected from six NHS Hospital Trusts in South West England. The area covered by these Trusts has one of the lowest population densities in the country, with people living across a mix of coastal communities, isolated rural areas, and urban centres.[18]

Data for EMS calls were collected from two NHS ambulance service Trusts using the two different Clinical Decision Support Systems used in the UK: the Advanced Medical Priority Dispatch System (AMPDS) (Salt Lake City, USA) and NHS Pathways (NHS Digital, Leeds, UK). Data from calls triaged by AMPDS were collected from the South Western Ambulance Service NHS Foundation Trust (SWASFT), while data from calls triaged by NHS Pathways were collected from the North East Ambulance Service NHS Foundation Trust (NEAS). AMPDS has been in use in the United States since 1978 and is used by emergency services in 59 countries, including roughly half of ambulance service Trusts in the UK.[17] NHS Pathways is used by the other UK ambulance trusts and is overseen by a National Clinical Governance Group (NCGG), which is hosted by the Royal College of General Practitioners and is made up of representatives from UK medical royal colleges.[17]

Patient and public involvement (PPI)

A dedicated PPI group guided this work from its inception, supporting the development of research questions and study design. The group consisted of four women and two men who had experience of BBA. Experiences were varied and represented ethnic, cultural and geographical diversity. Over the course of the project, the PPI group reviewed and refined participant-facing documents for stakeholder focus groups, contributed to data synthesis, and advised the team on the approach to recruitment and dissemination.

Study design

This multi-methods study consisted of both qualitative and quantitative data with two phases. Phase A included an analysis of hospital data and Phase B included content analysis of EMS calls, followed by thematic analysis of focus groups with NHS staff and patients.

The two phases of this study were funded as one piece of work and linked through their focus on inequality. Whilst we wanted to use Phase A of this work to examine potential inequalities surrounding BBA births in the South West of England, we also wanted to examine the accessibility of EMS call-handler advice regarding neonatal hypothermia. This would help to identify potential opportunities to improve this advice by ensuring we were aware of the populations most at risk, and providing the EMS call-handlers with accessible and easily understandable information.

Phase A: Neonatal hypothermia on arrival at hospital

Anonymised extracts from routinely collected, non-identifiable data (hospital neonatal records for BBA babies), were provided by participating Trusts for a three-year period (January 2018-January 2021). These were compared with National Maternity Data from the same geographical area o understand which groups are most likely to experience BBA and how this would relate to hypothermia and neonatal outcomes recorded by Hospital Trusts (including admission to a neonatal unit (NNU), length of hospital stay, and treatment provided).

Recruitment

Hospital Trusts were eligible to take part if they were covered by the South West Academic Health Science Network (AHSN).[18] The South West AHSN presented information about the study in a

For peer review only

number of online events and invited Trusts to email an expression of interest to the study lead to take part. Participating Trusts were offered administration costs to collect and anonymise data.

Data collection

An Excel spreadsheet (Microsoft, Redmond USA) was created to standardise the information provided by Hospital Trusts. Data points selected for inclusion were all demographic characteristics for the mother and baby, and all care and outcome information related to neonatal temperature, that were available from the maternity notes held by the Hospital Trusts. The spreadsheet was piloted and refined by midwives and neonatal nurses at one of the participating Trusts, before circulating. Members of maternity and neonatal staff at each participating Trust searched their records for cases of BBA, uploaded anonymised information to the spreadsheet, and returned it to the study lead. Records were included if they related to a live birth in the prehospital setting where the baby was brought to hospital by EMS (ambulance staff).

Analysis

Due to the small number of BBA cases recorded over the time period specified above, we did not do an in-depth statistical analysis with statistical testing. As such, demographic characteristics of the mothers (booking status, parity, safeguarding status) and characteristics of the birth (gestation, temperature on admission, treatment) are reported using descriptive statistics.

Phase B: 999 call-handler advice on neonatal temperature management

Analysis of current advice

Two UK NHS EMS provider organisations (Ambulance Trusts) provided anonymised audio-recordings of calls relating to BBA that were analysed to investigate the temperature management advice given by EMS call-handlers during emergency calls.

Data collection

All EMS calls relating to BBA between January 2021 and January 2022 were searched by the Clinical Information and Records (CIR) teams at the two participating EMS provider organisations (ambulance services). The team used an EMS diagnosis code of 'normal delivery' (a single live birth with no obstetric complications) to find relevant calls. Calls were listened to by the CIR team to check eligibility, and 30 eligible calls (15 from each ambulance service) were selected at random from postcodes with varying levels of deprivation (Figure 1). Deprivation was determined by the team through the lower layer super output area of the incidents, which gave an overall index of multiple deprivation (IMD) score for each call. Anonymised audio-recordings of the calls were then transferred to the study lead and transcribed by a University-approved transcription company.

Analysis

Data were analysed by LG and AJ using content analysis, and findings were used to develop an audiovisual presentation to stimulate discussion during focus groups (Figure 1).

Staff and patient views

Focus groups were conducted with NHS staff and patients to investigate opportunities to improve the temperature management advice given during EMS calls.

Recruitment

A purposive sampling technique was adopted to ensure rich information about the topic.[19] Participants were eligible to take part in staff focus groups if they were a practicing member of NHS staff with experience of BBA (e.g., paramedic, midwife, neonatal nurse/doctor, EMS call-handler). Participants were eligible to take part in patient focus groups if they had experienced BBA (e.g., as a parent or caller to EMS).

For peer review only - http://bmjopen.bmj.com/site/about/guidelines.xhtml

Recruitment adverts were circulated using social media and collaborating organisations (South West AHSN, Stillbirth and Neonatal Death Society (SANDS) charity), and participants were reimbursed with a £25 e-voucher for their time. Eligible participants were invited to contact LG to join a focus group. Potential participants were emailed a participant information sheet, privacy notice and consent form. Verbal confirmation of consent was audio-recorded at the start of each focus group. We aimed to recruit 30-40 participants, guided by information power with a narrow aim and specific study population.[20]

Data collection

Four focus groups were held via Teams (Microsoft, Redmond USA) with NHS staff, facilitated by LG. The content and structure of BBA calls were discussed in relation to their ideas on best practice temperature management advice, in response to findings from the content analysis of EMS calls, using the presentation described above (Figure 1).

Themes identified during these focus groups were used to create a second audiovisual presentation to facilitate focus group discussions with members of the public (Figure 1). Five focus groups were held via Teams and focused on the content and accessibility of the advice given during EMS calls, as well as the suggestions put forward by NHS staff.

Analysis

Focus groups were video-recorded and transcripts generated by Teams. Transcripts were coded and analysed by LG using thematic analysis. Findings were synthesised to create a set of recommended adaptations to the current advice given by call-handlers using EMS triage systems. These recommended adaptations were sent to focus group participants and the PPI group for final review and refinement.

RESULTS

Phase A: Neonatal hypothermia on arrival at hospital

217 babies were conveyed to the 6 hospital Trusts by the ambulance service during the above time-period (live births ≥24 weeks). There were 33 records (15%) with no neonatal arrival temperature documented.

Of those with a temperature recorded, 35% (64/184) were hypothermic on arrival at hospital. Table 1 gives a breakdown of these recorded temperatures according to World Health Organization (WHO) classifications of core body temperature for newborns.[8]

Table 1: Neonatal temperature on arrival at hospital

**WHO classification**	**Temperature range**	**Number of babies (%) n = 184**	**Implication for outcomes/care (WHO)**
Hyperthermia	37.6	1 (1%)	Cause for concern
Normal range	36.5 – 37.5°C	119 (65%)	N/A – normal care
Cold stress (mild hypothermia)	36 - 36.4°C	48 (26%)	Cause for concern
Moderate hypothermia	32 - 35.9°C	14 (8%)	Danger; warm baby
Severe hypothermia	<32°C	2 (1%)	Outlook grave, skilled care urgently needed

For peer review only - http://bmjopen.bmj.com/site/about/guidelines.xhtml
For peer review only

Inequalities in chances of having a BBA

Hospital data from this study were compared to South West data from the National Maternity Data Set for 2021-2022.[21]

Some characteristics were more prevalent in the data for BBAs compared with local data for all births (Table 2). This included safeguarding concerns documented at booking, previous live births, and late booking (past 13 weeks). A definition of all characteristics is provided in Supplemental file 1.

Table 2. Comparisons made between BBA data and overall data for the South West

Characteristic		BBA data Number (%)	Overall South West data Number (%)
**Safeguarding concerns documented at booking**		**n = 217**	**n = 48900**
	Yes	47 (22%)	4180 (8%)
	No	170 (78%)	43425 (89%)
	Missing data	0 (0%)	1295 (3%)
**Previous live birth(s)**		**n = 217**	**n = 48905**
	Yes	185 (85%)	26875 (55%)
	No	32 (15%)	19125 (39%)
	Missing data	0 (0%)	2905 (6%)
**Gestation at booking***		**n = 217**	**n = 48900**
	Early booker (up to 12+6 weeks)	179 (83%)	46020 (94%)
	Delayed booker (13 to 19+6 weeks)	20 (9%)	1835 (4%)
	Late booker/not booked (≥20 weeks)	14 (6%)	935 (2%)
	Missing data	4 (2%)	110 (0.2%)
**Mother's age at booking**		**n = 217**	**n = 48900**
	Under 20	7 (3%)	1410 (3%)
	20 to 29	90 (41%)	20,000 (41%)
	30 to 39	108 (50%)	25,725 (53%)
	40 or over	11 (5%)	1765 (4%)
	Missing data	1 (0.1%)	0 (0%)

*Booking classifications are presented as defined by The National Institute for Health and Care Excellence (NICE)[22]

Whilst we set out to compare the ethnicity of women experiencing BBA with the overall South West data, this was prevented by the use of different ethnicity categories in the two data sets. For those experiencing BBA recorded ethnicity was: British European 94%, East European 2%, Other 2%, Indian 0.5%, North European 0.5%, South African 0.5%, South European 0.5%. For those in the South West data set ethnicity was: White 84%, Asian or Asian British 3%, Black or Black British 2%, Mixed 2%, Any other ethnic group 2%. We were also unable to consider social deprivation, as we had hoped to do, as we were only provided with the first half of patients' postcodes by the Trust. This was done to maintain anonymity of patient data.

For peer review only - http://bmjopen.bmj.com/site/about/guidelines.xhtml

Inequalities in neonatal hypothermia/poor outcomes

Hypothermia on arrival at hospital was more prevalent for pre-term babies, babies born to mothers who had not had a previous live birth, and babies born to mothers who reported a disability or safeguarding concern at booking (Table 3).

Table 3: Characteristics of mothers and babies, by neonatal temperature on arrival at hospital, n = 184

**Characteristic of baby/mother**		**Hypothermia <36.5°C n = 64 Number (%)**	**Normothermia 36.5-37.5°C n = 119 Number (%)**
Prematurity	Pre-term (<37 weeks)	9 (14%)	2 (2%)
	Term (37+ weeks)	54 (84%)	116 (97%)
	Unknown	1 (2%)	1 (1%)
Previous live birth	Yes	48 (75%)	109 (92%)
	No	16 (25%)	10 (8%)
Disability reported at booking	Yes	6 (9%)	2 (2%)
	No	58 (91%)	115 (97%)
	Unknown	0 (0%)	2 (2%)
Safeguarding concerns reported at booking	Yes	19 (30%)	20 (17%)
	No	45 (70%)	99 (83%)
Gestation at booking	Early booker (up to 12+6 weeks)	51 (80%)	101 (85%)
	Delayed booker (13 to 19+6 weeks)	6 (9%)	13 (11%)
	Late booker/unbooked (≥20 weeks)	6 (9%)	3 (2%)
	Unknown	1 (2%)	2 (2%)
Mother's ethnicity	British European	58 (91%)	114 (95%)
	East European	3 (4.5%)	0 (0%)
	Indian	0 (0%)	1 (1%)
	North European	1 (1.5%)	0 (0%)
	South African	1 (1.5%)	0 (0%)
	South European	0 (0%)	1 (1%)
	Other	1 (1.5%)	4 (3%)
Mother born in UK	Yes	57 (89%)	112 (94%)
	No	6 (9%)	5 (4%)
	Unknown	1 (2%)	2 (2%)
Model of care	Consultant-led	25 (39%)	48 (40%)
	Midwifery-led	37 (57%)	70 (59%)
	Unknown	2 (3%)	1 (1%)

For peer review only - http://bmjopen.bmj.com/site/about/guidelines.xhtml

Mother's attendance at antenatal appointments	All attended	52 (81%)	88 (74%)
	Not all attended	12 (19%)	25 (21%)
	Unknown	0 (0%)	6 (5%)
Characteristic of baby/mother		<36.5°C<br>n = 64<br><br>Median (IQR)	≥36.5°C<br>n = 119<br><br>Median IQR
Mother's age at booking		28 (23, 34))	31 (27, 35))

Babies with neonatal hypothermia on arrival at hospital were more likely to be admitted to a Neonatal Unit (NNU) or receive advanced care (33% vs 19%). Advanced care was classified as any care needed in addition to 'normal newborn care', for example the provision of oxygen or intravenous (IV) medication.[23]

Of the hypothermic babies needing advanced care/admission to an NNU, a slight majority were born at term (11/21; 52%), one had a diagnosis of sepsis confirmed (5%), and just over half (11/21; 52%) were either moderately or severely hypothermic on arrival.

Seven (11%) hypothermic babies had an extended length of stay in hospital (>7 days) whereas none of the warm babies did. Due to the small sample size we were unable to perform in-depth statistical analysis to control for potential confounding variables. However, most (6/7, 86%) of the babies with an extended length of stay were also preterm. More treatment was given to hypothermic babies (glucose: 8% vs 1%; radiant heat source: 20% vs 3%; and incubator: 11% vs 3%).

Phase B: EMS call-handler advice on neonatal temperature management

Participant characteristics

Eighteen NHS staff took part in the four staff focus groups. Most participants were female (13/18, 72%). Participants' job titles comprised: midwives, paramedics, call-handlers, learning development officer, neonatal nurses, neonatal doctors, advanced care practitioners.

Twenty-two members of the public who had experience of prehospital birth took part in five focus groups. Most participants were female (18/22, 82%). Participants had a range of experiences, representing diverse geographical regions, areas of deprivation, and prehospital birth experience (e.g., at home, in the car, outside).

Five main themes were identified as affecting neonatal temperature management during EMS calls. These included: 1) the importance placed on neonatal temperature; 2) advice on where the baby should be placed following birth; 3) advice on how to keep the baby warm; 4) the timing of temperature management advice; and 5) the clarity and priority of instructions. Illustrative quotes are provided in Table 4.

Table 4: Illustrative quotes from qualitative data from focus groups

Quote no.	Focus Group	Quote
1	Public Focus Group 2	Nothing was mentioned about keeping the baby warm and to be honest, I had no idea that that's something that I should be doing.

For peer review only - http://bmjopen.bmj.com/site/about/guidelines.xhtml

2	Public Focus Group 2	When I delivered my daughter, their concern was mainly on delivering the placenta. It was my mum that had suggested that I should take my baby into my jumper that I was wearing to keep her warm because it was a cold morning. It was only until they realised that they couldn't deliver my placenta that they told me to get the blanket out and then wrap my daughter.
3	Staff Focus Group 2	You'll find that people want something to do. They want to focus in on something. And if you were to give that information around the importance of thermal regulation 'keeping that baby warm is really important now/ When that baby's born, we really want to keep that baby warm.' I think that would be really useful for that person who's trying to do something positive.
4	Staff Focus Group 2	Often when we're reviewing cases, we'll kind of note that as we kind of arrived on scene, the baby is on the floor and the mother's just in shock and the babies on a cold floor and it's drafty.
5	Public Focus Group 4	What we found really really confusing is that all the way through we were advised skin-to-skin, and don't do anything with the cord - keep that connected as long as possible. But then when it came to the actual 999 caller, it was totally different advice and it was 'keep the baby on the back'…Having that totally conflicting advice put my partner in the position where he was like 'I'm totally confused'.
6	Public Focus Group 4	When the baby did come out, she was just lying on the bed on her back. And I instinctively wanted to take her and put her on my chest like I did before with the midwives, and the instruction was to leave her on her back. I wasn't allowed to pick her up.
7	Public Focus Group 2	I was in a shopping mall, and there were lots of people around me. Lots of people looking and crowding. So I couldn't feel like I could do skin-to-skin. I was in panic and the security guy was a man. I'm sad I didn't get to do it, but it was too much stress.
8	Public Focus Group 4	I completely did not think straight in that moment at all…I was just a bit of an idiot, to be honest. And so when he came out, I didn't think about keeping him warm…I don't think you can rely on people thinking straight in that moment. … I think sometimes like I said, stating the obvious is quite useful in that kind of scenario.
9	Public Focus Group 4	We had quite a long wait before the midwife came. My husband was really panicking…but actually if someone had said to him 'move to a warm room' or 'go and put the heating on, if you haven't got it on already', he would have done that straight away.
10	Staff Focus Group 1	The gap should be used to paraphrase back to them. Just saying, you know…like 'dry, warm and wrapped'. Just keeping it simple repeating it back to them, showing that they understand the instruction for…however long it takes [the paramedics] to finally get there.
11	Public Focus Group 4	If it seems like it's a rushed labour, then perhaps maybe focus on what's going on at hand. And then perhaps maybe give the [temperature] advice thereafter like step by step... Whereas if it's maybe not as quick, maybe strike some sort of conversation with the person that's with the mother and say 'ok, when the baby does come out, remember to keep the baby warm.
12	Public Focus Group 1	If they can [provide warming advice] straight away if they've got time, then yeah, do it again also in between [gaps in the conversation]. If they've got time waiting for the ambulance, just mention it again. Just

For peer review only - http://bmjopen.bmj.com/site/about/guidelines.xhtml

		maybe check, check baby's chest or, you know, make sure it's warm or just little things like that because you do, you do forget.
13	Staff Focus Group 4	Babies being born in the sac is really rare. Usually it's you know, contractions, suddenly the water's gone, and the baby follows it quite quickly with these mums…So I don't think that should even be in there really because that might even worry mums like 'well, should there be a sac? Is it wrong that they're not in a sac? Oh my goodness me, you know, where's the sac?' And then start looking for a sac.

Theme 1: The importance placed on neonatal temperature

Although call-handlers from both triage systems repeated the instruction to 'keep mum and baby warm' at several points during the EMS call, participants noted that the importance of neonatal temperature was not explicitly addressed. This was of concern to participants who had experienced a BBA, who said that they were unaware of how quickly babies could become hypothermic in the prehospital setting, or how this could affect neonatal outcomes. It was noted by participants that antenatal information did not provide much information on neonatal hypothermia so they were unprepared for it (Q1).

Most participants reported that call-handlers had focused their advice and instructions on the person giving birth (e.g., determining whether the placenta had been delivered/whether there was any bleeding), and that limited attention had been given to the baby once it was established that they were breathing. Some reported that their baby had become hypothermic following their BBA and expressed frustration that it had not been made a priority or been communicated how important it was to keep their baby warm (Q2).

All participants felt that the importance of temperature could be further highlighted. Participants who had experienced prehospital birth suggested that this should be communicated in clear and simple language. NHS staff suggested that giving the caller the 'job' of keeping the baby warm could help emphasise this importance and increase the chance of temperature management being performed well (Q3).

Theme 2: Where the baby should be placed following birth

The two triage systems differed in their instruction on where the baby should be placed following the birth. The AMPDS triage system states that callers should 'keep the baby between the mother's legs and level with her bottom', while NHS Pathways states that callers should 'place the baby on the mother's chest or tummy'.

Participants expressed frustration at the instruction to place the baby between the mother's legs and felt this could be very detrimental to temperature management, especially if the mother was in a room with a cold floor, such as a bathroom or kitchen (Q4).

Participants who had received this advice following prehospital birth said that it had caused confusion, as it contradicted what they had been taught antenatally (i.e., skin-to-skin contact following birth) (Q5).

For those who had given birth in a car, this advice had proven especially challenging. One participant noted that by trying to keep the baby between her legs her husband had had to open all the car doors, which had potentially contributed to her baby being hypothermic on arrival at hospital.

For peer review only - http://bmjopen.bmj.com/site/about/guidelines.xhtml

Some participants spoke of not being 'allowed' to pick their baby up following the BBA and to keep their baby on its back on the bed/floor until the paramedics arrived (Q6).

Skin-to-skin contact with the mother was put forward by NHS staff as the optimal practice for neonatal normothermia following prehospital birth. There was some discussion amongst staff regarding the importance of maintaining a clear airway during skin-to-skin contact, and it was acknowledged that regular prompts would need to be given by call-handlers to check that the baby's nose and mouth could be seen. Participants also emphasised the need for clear and simple instructions to be given by call-handlers on how to conduct skin-to-skin contact effectively, including checking to see if the mother was warm themselves.

Most participants said that skin-to-skin contact would have been practical in the circumstances surrounding their BBA. Some circumstances were seen as potentially problematic for skin-to-skin contact, for example, if the cord was too short, they were too anxious, or were not confident in handling the baby (e.g., first-time parents). Some participants expressed concerns over privacy regarding skin-to-skin contact and said that they would not feel confident doing this in a public place or if they thought male paramedics might attend (Q7).

Staff also acknowledged that there may be cases where skin-to-skin contact was not appropriate or possible, for which staff suggested thorough drying and wrapping of the baby before placing them on someone warm.

Ensuring that the baby was put in the optimal place was discussed by participants as particularly important in the context of long wait times for ambulances and the "cost of living crisis". Participants noted that people could potentially be waiting for hours for an ambulance in a cold house, so ensuring the baby is kept as warm as possible should be a priority when advising callers where to place the baby following birth.

Theme 3: How to keep the baby warm

Neither of the two triage systems contain specific advice on how to keep the baby warm, although some call-handlers did add some advice themselves during calls (e.g., suggesting the use of a hat). Generic advice of 'keep mum and baby warm' was given by each pathway, and callers were also told to 'cover the baby's head, but not its face' and to 'use towels and blankets to keep the baby warm'.

During focus groups with NHS staff, participants expressed a preference for warming advice to be more specific and targeted. Suggestions included advising skin-to-skin contact (as above), putting a hat on the baby, considering possible changes to the environment (e.g., closing any windows, moving off cold floors, putting the heating on), and emphasising the need to dry the baby thoroughly and replace wet towels with dry ones. Participants also suggested adding an instruction for callers to check the warmth of the baby by touching the skin on the chest/back, to see if warming measures were working.

The specific advice put forward by staff was viewed positively in focus groups with those who had experienced a BBA. Many suggested that they had experienced stress and anxiety during their BBA, and that this had caused them to 'lose all common sense' or be unable to think rationally. As such, the instruction to 'keep the baby warm' was viewed as too generic and ambiguous by participants, who felt that clear, practical instructions would be easier to follow and would help them to feel more in control of the situation (Q8).

For peer review only - http://bmjopen.bmj.com/site/about/guidelines.xhtml

Most participants felt that all warming measures suggested by staff would have been practical in the circumstances of their BBA, and that alternatives would have been easily considered if not (e.g., using an item of clothing instead of a blanket/towel). Participants did acknowledge, however, that some warming measures may not be practical in all situations, and that callers should be advised that each warming measure should be carried out 'if possible'. Participants also noted that listing all warming measures may not be appropriate if the woman was calling for herself and had no-one around to assist her.

Of the measures suggested by staff, potential changes to the environment were emphasised by participants as something that could have considerably influenced their baby's temperature. Participants suggested that simple advice such as moving to a warmer room would have given them a sense that they were positively impacting the situation and helped relieve some of the anxiety and panic (Q9).

Theme 4: Timing of temperature management advice

Another key theme about the content and structure of the EMS triage systems was the timing of neonatal temperature management advice. While call-handlers from both pathways focused this advice predominantly after the baby was born, long gaps were identified in some calls while call-handlers waited for a part of the baby to be seen by the caller. NHS staff suggested that these gaps could be used to emphasise the importance of temperature, and to give advice on warming the environment if appropriate. Gaps in the conversation were also identified during periods of time where call-handlers had completed the triage algorithm and were waiting for paramedics to arrive. These gaps were often filled with small-talk. Staff suggested that this would be an appropriate time to give specific warming advice, and for call-handlers to check that essential warming measures had been completed (Q10).

Participants who had experienced a BBA felt that warming advice should be given as soon as possible, but without interrupting vital instructions. Some participants felt that the woman should be the priority and focus of calls before the baby was born, but that a brief mention of the importance of temperature would be helpful, and that focus should then shift to keeping the baby warm after birth (Q11).

Participants agreed with NHS staff that once the triage algorithm was complete, any additional time spent on calls waiting for the ambulance should be used to provide specific warming advice and to check it had been followed appropriately. Participants felt that it would be helpful to be reminded of all the steps they could take to keep their baby warm at any possible opportunity (Q12).

Focus group participants also discussed advice that could be given antenatally to prepare them for neonatal temperature management in the prehospital setting. Suggestions included an antenatal leaflet on the importance of neonatal thermoregulation, a reminder at antenatal appointments, and/or advice to pack a blanket in their hospital bag (in case of birthing in the car).

Theme 5: Clarity and priority of instructions

The clarity and priority given to instructions was discussed by participants in both focus groups. Several instructions were highlighted by participants as potentially confusing or distracting, and some felt that the timing and priority given to these instructions could delay neonatal temperature management. For example, calls from the NHS Pathways algorithm included instructions for the caller to 'wipe or peel away the sac' before drying and wrapping the baby. This frequently caused confusion to callers, as none of the babies were born in their sac and meant that drying and wrapping instructions were delayed while the call-handler and caller discussed the nature of the sac.

For peer review only

NHS staff suggested that the previous instruction to 'wipe the baby's nose and mouth' should be sufficient to cover any issues with the sac and suggested that this instruction be removed or altered to avoid confusion. Participants from both sets of focus groups suggested that once the baby was born, only essential instructions should be given before warming advice, as to avoid any confusion or distraction (Q13).

Other instructions questioned by NHS staff included the advice to get towels and blankets to 'protect the bed or floor', and the caller being directed to wash their hands once a part of the baby could be seen.

DISCUSSION

This is the first study, to our knowledge, to investigate the temperatures of babies arriving at hospital following a prehospital birth (birth before arrival; BBA) in South West England. We found that 35% of babies born prehospital arrived at hospital hypothermic (<36.5°C), and 25% of these were classed as 'moderately' or 'severely' hypothermic, with warming needed urgently to prevent poor outcomes. These findings are in line with those of a study in Finland, which found hospital diagnosis of neonatal hypothermia to be recorded in 53% of babies born out of hospital, compared to only 0.7% of babies born in hospital.[4] Our findings also concur with research from France, which found that 56% of babies born outside of hospital were hypothermic on arrival at hospital,[16] and that neonatal hypothermia was associated with increased rates of NICU admission.[5] Although our findings suggest better rates of neonatal normothermia following BBA than literature from other areas, 35% remains a significant proportion of babies arriving at hospital hypothermic. This could potentially be improved through better availability of optimal equipment such as transwarmers and accurate temperature monitoring devices, further training, increased awareness amongst ambulance staff,[10] and improvements in EMS call-handler advice.

We found that particular babies may be more at risk of hypothermia on hospital arrival following BBA. These include pre-term babies, babies of first-time mums, babies of women where there are safeguarding concerns, and babies of women who reported disabilities at their booking appointment. While prematurity and low birthweight are established risk factors for hypothermia in newborns generally,[24,25] there is a paucity of previous literature exploring the other potential risk factors we identified. It may be that where BBA occurs these factors make it harder for ambulance staff to effectively maintain the temperature of the newborn. This may be due to a lack of maternal awareness of the risks of hypothermia prior to EMS arrival and the nature of the birthing environment which may be unprepared for delivery. The findings from this study suggests that there is a lack of consistent advice provided to EMS callers prior to the arrival of the ambulance that does not effectively support temperature management at birth. While our study is limited by the small sample size and focused geographical location these preliminary associations suggest hypotheses and provide the basis for future research, as well as helping NHS services to identify babies who may require additional warming and support following a BBA. Further work should replicate and expand our study to consider areas of high population density, with a larger sample size to enable a more detailed statistical analysis.

Our findings suggest that there may be some women in South West England who are more likely to experience BBA, which previous research suggests may result in poorer outcomes for their babies.[4,5] These include women with safeguarding concerns, women who book their pregnancies with a health professional later than 13 weeks gestation, and those who have had previous babies. Multiparity, late booking and/or lack of antenatal care are known to be associated with BBA from previous literature,[2,4,12,26] however safeguarding concerns at booking has not been identified previously. Safeguarding concerns might include denial of pregnancy and maternal substance

For peer review only - http://bmjopen.bmj.com/site/about/guidelines.xhtml

misuse. It seems likely that these factors are associated with delays in identifying the onset of labour and presenting to maternity services. It is also well known that the average duration of labour in multiparous women is shorter than it is in nulliparous women.[27] Due to the small sample size of this study, caution should be exercised when interpreting results, but this finding may help maternity services identify and support women who are more likely to experience BBA. Despite receiving information on mothers' ethnicity from our BBA data, we were unable to explore any associations, as local ethnicity data were recorded differently to the National Maternity Data Set.[21] Work is ongoing to resolve this issue.

This study is the first to investigate neonatal temperature management advice provided by EMS call-handling triage systems. EMS dispatching is complex, and a call-handler must triage the call to inform the prioritisation of resource dispatch whilst also providing first aid advice prior to the arrival of an EMS resource (pre-arrival advice).[28] Our research identifies issues with unclear and contradictory pre-arrival advice during UK EMS calls regarding BBA, and suggests five themes that could influence neonatal thermal care during these calls. NHS staff and members of the public made several recommendations for changes to the script to address potential barriers to good thermal care, based on these themes. These changes have already been implemented in NHS Pathways, and similar changes are pending approval in AMPDS.

Although this is the first study to investigate neonatal temperature management advice given by EMS call-handlers, increasing attention is being paid to call-handler advice and interactions with patients,[29] especially during maternity cases.[17] Indeed, the Healthcare Safety Investigation Branch (HSIB) report into 'Maternity pre-arrival instructions by 999 call handlers'[17] similarly found that the two triage systems in England provide different pre-arrival instructions for birth, associated with different implications and risk. There is a growing body of evidence investigating the call-handler and caller interaction during EMS calls,[29] and this research should be considered in any proposed changes to emergency maternity pre-arrival advice. While much of the research into call-handler and caller interaction during the EMS call has focused on the out-of-hospital cardiac arrest (OHCA) scenario, these findings can be used to inform emergency maternity pre-arrival instructions and align with our own findings. For example, simplified instructions have been associated with a shorter interval between instruction and first chest compression in telephone cardiopulmonary resuscitation instruction in OHCA.[30] Future research could usefully investigate the design, implementation, and impact of emergency maternity pre-arrival advice.

CONCLUSION

The findings of this study suggest there may be certain women who are more likely to have a BBA in South West England and certain babies who are more likely to be hypothermic on arrival at hospital following a BBA. Findings also suggest potential areas for improvement to EMS call-handler advice regarding neonatal temperature management. Future work in this area has the potential to reduce the number of babies arriving at hospital hypothermic and improve longer term outcomes, thereby addressing health inequalities by disproportionately benefitting underserved groups.

LEGENDS

Figure 1: Flowchart to show the process of how call-handler data and focus group data were identified, screened, and analysed.

For peer review only - http://bmjopen.bmj.com/site/about/guidelines.xhtml

STATEMENTS

Acknowledgments
We would like to thank the NHS staff and members of the public who took part in focus groups for this evaluation, as well as the NHS Hospital and Ambulance Service Trusts who provided data.

We would also like to thank: Alice Jennings from UWE Bristol, for supporting the collection of hospital data and contributing to analysis of EMS calls; Sally Hedge from the South West AHSN for supporting and promoting this work; and the members of our Patient and Public Involvement Group for their invaluable input and contributions to this study: Tasha Cooper, Farzana Kausir, Poonam Barnes, Louise Walker, Simon Walker and Arif Hoque.

Finally, we would like to thank the funder of this project, the South West AHSN, and the study sponsor, UWE Bristol. This work was carried out by members of the Research in Emergency Care, Avon Collaborative Hub (REACH Bristol).

Patient and Public Involvement
Patients and/or the public were involved in the design, or conduct, or reporting, or dissemination plans of this research. A dedicated PPI group guided this work from its inception, supporting the development of research questions and study design. The group consisted of four women and two men who had experience of BBA. Experiences of the team were varied and represented ethnic, cultural and geographical diversity. Over the course of the project, the PPI group reviewed and refined participant facing documents for stakeholder focus groups, contributed to data synthesis, and advised the team on the approach to recruitment and dissemination. They also read and commented on the draft of this manuscript. Findings were shared with both the PPI group and study participants via email, presentations, and national conferences.

Patient consent for publication
Not required

Ethics committee approval
Ethics committee approval for this service evaluation was granted by the University of the West of England (UWE Bristol) Faculty of Health and Applied Sciences Research Ethics Committee (HAS.22.01.061). Participants gave informed consent to participate in the study before taking part.

Provenance and peer review
Not commissioned; externally peer reviewed.

Contributorship statement
LG was the Chief Investigator/guarantor with overall responsibility for the study, and participated in study conception, design and coordination, performed and coded the focus groups, analysed the data and drafted the manuscript. HT participated in study conception, design and interpretation of the results, and supported quantitative data analysis. GM, KK, EB, AB, TD, RO, HM, RMC, NM, SV and JB participated in study conception, design and interpretation of the results. All authors were responsible for the critical revision of the manuscript for publication and approved the final version to be published.

Competing interests
None declared

For peer review only

Funding

The study was funded by the South West Academic Health Science Network (AHSN) through its Perinatal Equity programme.

Data sharing statement

Data are available upon reasonable request.

REFERENCES

1. Office for National Statistics. Vital statistics in the UK: births, deaths and marriages - Office for National Statistics. Published 2019. Accessed January 30, 2024. https://www.ons.gov.uk/peoplepopulationandcommunity/populationandmigration/populationestimates/datasets/vitalstatisticspopulationandhealthreferencetables

2. Loughney A, Collis R, Dastgir S. Birth before arrival at delivery suite: Associations and consequences. *Br J Midwifery*. 2006;14(4):204-208. doi:10.12968/bjom.2006.14.4.20786

3. Snowden JM, Tilden EL, Snyder J, Quigley B, Caughey AB, Cheng YW. Planned Out-of-Hospital Birth and Birth Outcomes. *N Engl J Med*. 2015;373(27):2642-2653. doi:10.1056/NEJMsa1501738

4. Ovaskainen K, Ojala R, Gissler M, Luukkaala T, Tammela O. Out-of-hospital deliveries have risen involving greater neonatal morbidity. *Acta Paediatr*. 2015;104(12):1248-1252. doi:10.1111/apa.13117

5. Javaudin F, Hamel V, Legrand A, et al. Unplanned out-of-hospital birth and risk factors of adverse perinatal outcome: findings from a prospective cohort. *Scand J Trauma Resusc Emerg Med*. 2019;27(1):26. doi:10.1186/s13049-019-0600-z

6. Nguyen ML, Lefèvre P, Dreyfus M. [Maternal and neonatal outcomes of unplanned deliveries]. *J Gynecol Obstet Biol Reprod (Paris)*. 2016;45(1):86-91. doi:10.1016/j.jgyn.2015.02.002

7. Chitty H, Wyllie J. Importance of maintaining the newly born temperature in the normal range from delivery to admission. *Semin Fetal Neonatal Med*. 2013;18(6):362-368. doi:10.1016/j.siny.2013.08.002

8. World Health Organisation. Thermal Control of the Newborn: A Practical Guide. In: *Essential Elements of Obstetric Care at First Referral Level*. World Health Organization; 1993.

9. Perlman JM, Wyllie J, Kattwinkel J, et al. Part 7: Neonatal Resuscitation: 2015 International Consensus on Cardiopulmonary Resuscitation and Emergency Cardiovascular Care Science With Treatment Recommendations. *Circulation*. 2015;132(16 Suppl 1):S204-241. doi:10.1161/CIR.0000000000000276

10. Goodwin L, Voss S, McClelland G, et al. Temperature measurement of babies born in the pre-hospital setting: analysis of ambulance service data and qualitative interviews with paramedics. *Emerg Med J EMJ*. 2022;39(11):826-832. doi:10.1136/emermed-2021-211970

11. McClelland G, Burrow E, McAdam H. Babies born in the pre-hospital setting attended by ambulance clinicians in the north east of England. *Br Paramed J*. 2019;4(3):43-48. doi:10.29045/14784726.2019.12.4.3.43

For peer review only

12. Renesme L, Garlantézec R, Anouilh F, Bertschy F, Carpentier M, Sizun J. Accidental out-of-hospital deliveries: a case-control study. *Acta Paediatr Oslo Nor 1992*. 2013;102(4):e174-177. doi:10.1111/apa.12156

13. Diana L, Glaucia L, Adriana C, Israel F. Out-of-hospital deliveries: A case-control study. *Turk Pediatri Arsivi*. 2018;53(2):87-95. doi:10.5152/TurkPediatriArs.2018.6269

14. Moscovitz HC, Magriples U, Keissling M, Schriver JA. Care and outcome of out-of-hospital deliveries. *Acad Emerg Med Off J Soc Acad Emerg Med*. 2000;7(7):757-761. doi:10.1111/j.1553-2712.2000.tb02264.x

15. Adamson SK, Towell ME. THERMAL HOMEOSTASIS IN THE FETUS AND NEWBORN. *Anesthesiology*. 1965;26:531-548. doi:10.1097/00000542-196507000-00017

16. Javaudin F, Roche M, Trutt L, et al. Assessment of rewarming methods in unplanned out-of-hospital births from a prospective cohort. *Scand J Trauma Resusc Emerg Med*. 2020;28(1):50. doi:10.1186/s13049-020-00750-9

17. Health Services Safety Investigations Body. Investigation report: Maternity pre-arrival instructions by 999 call handlers. HSSIB. Published 2022. Accessed January 11, 2024. https://www.hssib.org.uk/patient-safety-investigations/maternity-pre-arrival-instructions-by-999-call-handlers/investigation-report/

18. Health Innovation South West. Health Innovation South West. Published January 17, 2024. Accessed January 30, 2024. https://healthinnovationsouthwest.com/

19. Patton M. *Qualitative Research & Evaluation Methods: Integrating Theory and Practice*. Sage Publications; 2014.

20. Malterud K, Siersma VD, Guassora AD. Sample Size in Qualitative Interview Studies: Guided by Information Power. *Qual Health Res*. 2016;26(13):1753-1760. doi:10.1177/1049732315617444

21. NHS Maternity Statistics, England - 2021-22. NHS Digital. Accessed January 30, 2024. https://digital.nhs.uk/data-and-information/publications/statistical/nhs-maternity-statistics/2021-22

22. Overview | Pregnancy and complex social factors: a model for service provision for pregnant women with complex social factors | Guidance | NICE. Published September 22, 2010. Accessed January 30, 2024. https://www.nice.org.uk/Guidance/CG110

23. Neonatal Transitional Care - A Framework for Practice (2017). British Association of Perinatal Medicine. Accessed January 30, 2024. https://www.bapm.org/resources/24-neonatal-transitional-care-a-framework-for-practice-2017

24. Miller SS, Lee HC, Gould JB. Hypothermia in very low birth weight infants: distribution, risk factors and outcomes. *J Perinatol Off J Calif Perinat Assoc*. 2011;31 Suppl 1:S49-56. doi:10.1038/jp.2010.177

25. Laptook AR, Salhab W, Bhaskar B, and the Neonatal Research Network. Admission Temperature of Low Birth Weight Infants: Predictors and Associated Morbidities. *Pediatrics*. 2007;119(3):e643-e649. doi:10.1542/peds.2006-0943

For peer review only - http://bmjopen.bmj.com/site/about/guidelines.xhtml

26. Bhoopalam PS, Watkinson M. Babies born before arrival at hospital. *Br J Obstet Gynaecol*. 1991;98(1):57-64. doi:10.1111/j.1471-0528.1991.tb10312.x

27. Ashwal E, Livne MY, Benichou JIC, et al. Contemporary patterns of labor in nulliparous and multiparous women. *Am J Obstet Gynecol*. 2020;222(3):267.e1-267.e9. doi:10.1016/j.ajog.2019.09.035

28. Møller TP, Jensen HG, Viereck S, Lippert F, Østergaaard D. Medical dispatchers' perception of the interaction with the caller during emergency calls - a qualitative study. *Scand J Trauma Resusc Emerg Med*. 2021;29(1):45. doi:10.1186/s13049-021-00860-y

29. Perera N, Finn J, Bray J. Can emergency dispatch communication research go deeper? *Resusc Plus*. 2022;9:100192. doi:10.1016/j.resplu.2021.100192

30. Leong W, Leong S, Arulanandam S, et al. Simplified instructional phrasing in dispatcher-assisted cardiopulmonary resuscitation – when 'less is more.' *Singapore Med J*. 2021;62(12):647-652. doi:10.11622/smedj.2020080

For peer review only

For peer review only - http://bmjopen.bmj.com/site/about/guidelines.xhtml

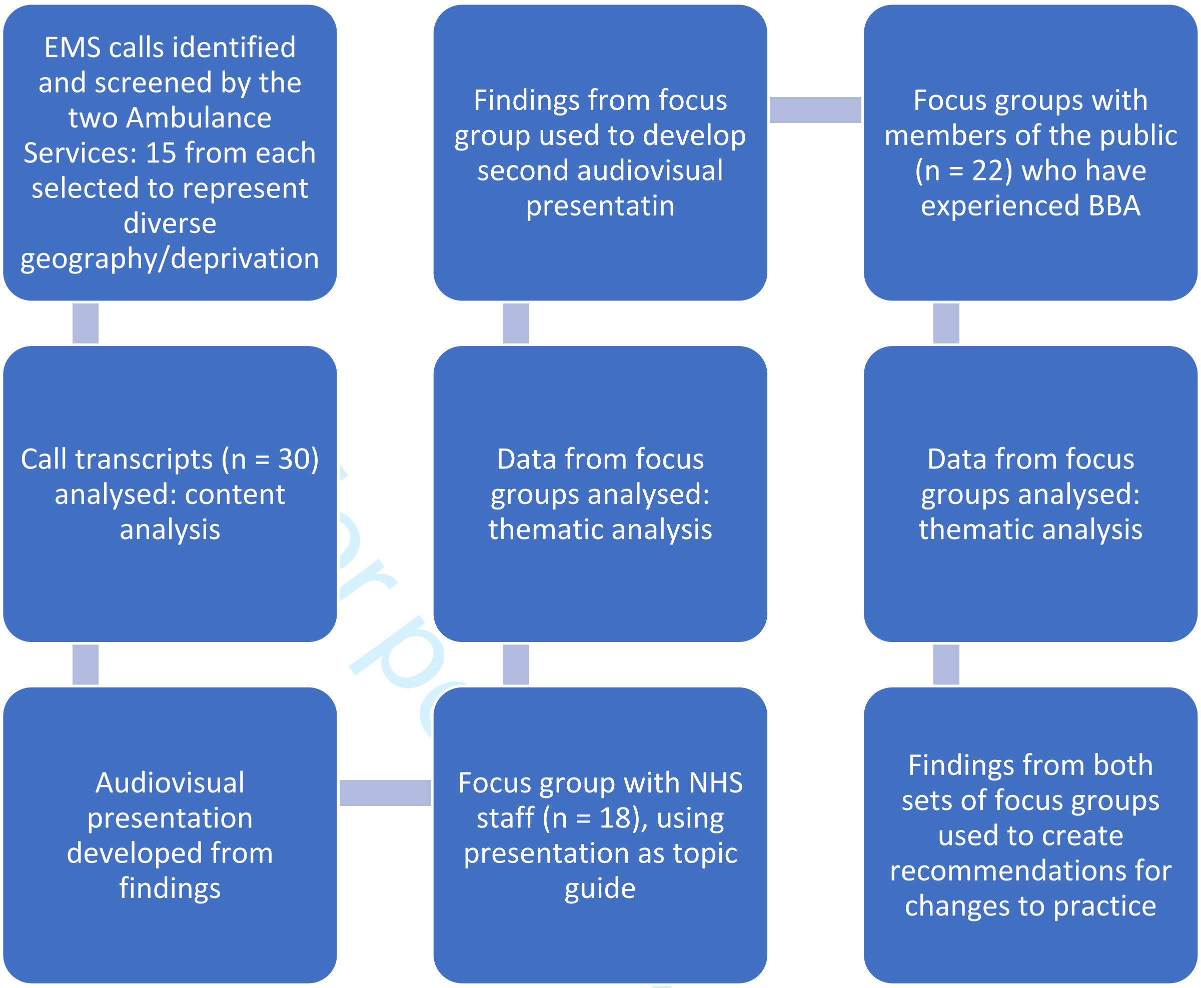

Figure 1: Flowchart to show the process of how call-handler data and focus group data were identified, screened, and analysed.

Supplemental File 1

Definitions of characteristics of mothers and babies

- Prematurity: recorded gestation at birth (weeks and days. Pre-term (<37 weeks); Term (37+ weeks).
- Previous live birth: self-reported by the mother and identified by the midwife.
- Disability reported at booking: any disability self-reported by the mother at the booking appointment.
- Safeguarding concerns reported at booking: any safeguarding concerns self-reported by the mother or identified by the midwife at the booking appointment.
- Gestation at booking: gestational age (weeks and days) recorded at the booking appointment.
- Mother's ethnicity: self-reported by the mother at the booking appointment.
- Mother born in UK: self-reported by the mother at the booking appointment.
- Model of care: recorded by the midwife.
- Mother's attendance at antenatal appointments: recorded by the midwife.
- Mother's age at booking: recorded by the midwife.

For peer review only

Supplementary Table 1: COREQ checklist

**Domain 1: Research team and reflexivity**		**Location in manuscript (Section, page no.)**
**Personal Characteristics**		
1. Interviewer/facilitator Which author/s conducted the interview or focus group?	LG	Methods - 5
2. Credentials<br>What were the researcher's credentials? E.g. PhD, MD	BSc, PhD	Title Page
3. Occupation<br>What was their occupation at the time of the study?	Senior Research Fellow in Emergency Care	Title Page
4. Gender Was the researcher male or female?	Female	N/A
5. Experience and training<br>What experience or training did the researcher have?	Experienced qualitative researcher.	Methods - 5
**Relationship with participants**		
6. Relationship established<br>Was a relationship established prior to study commencement?	No	N/A
7. Participant knowledge of the interviewer<br>What did the participants know about the researcher? e.g. personal goals, reasons for doing the research	Participants were briefed on the purpose of the study and were aware that ethical approval had been granted. Participants reviewed the participant information documentation prior to giving their written informed consent to be involved.	Methods - 5
8. Interviewer characteristics What characteristics were reported about the interviewer/facilitator? e.g. Bias, assumptions, reasons and interests in the research topic	No interviewer-related biases identified.	N/A
**Domain 2: study design**		
**Theoretical framework**		
9. Methodological orientation and Theory<br>What methodological orientation was stated to underpin the study? e.g. grounded theory, discourse analysis, ethnography, phenomenology, content analysis	Thematic analysis	Methods – 5
Participant selection		
10. Sampling How were participants selected? e.g.	Purposive sampling was used: recruitment adverts were circulated using social media and collaborating organisations	Methods - 5

For peer review only - http://bmjopen.bmj.com/site/about/guidelines.xhtml

11. Method of approach How were participants approached? e.g. face-to-face, telephone, mail, email	As above	Methods - 5
12. Sample size How many participants were in the study?	18 NHS staff and 22 members of the public	Results - 7
13. Non-participation How many people refused to participate or dropped out? Reasons?	There were no participants who subsequently refused to participate, withdrew consent or dropped out.	N/A
**Setting**		
14. Setting of data collection Where was the data collected? e.g. home, clinic, workplace	Data was collected and recorded via a Microsoft Teams meeting.	Methods - 5
15. Presence of non-participants Was anyone else present besides the participants and researchers?	No	N/A
16. Description of sample What are the important characteristics of the sample? e.g. demographic data, date	Most participants were female (72% of the NHS staff and 82% of the patients). For NHS staff, participants' job titles comprised: midwives, paramedics, call-handlers, learning development officer, neonatal nurses, neonatal doctors, advance care practitioners. For members of the public, participants had a range of experiences, representing diverse geographical regions, areas of deprivation, and prehospital birth experience (e.g., at home, in the car, outside).	Results – 7
**Data collection**		

For peer review only

17. Interview guide Were questions, prompts, guides provided by the authors? Was it pilot tested?	Focus group discussions were semi-structured using a PowerPoint presentation to guide discussion; the presentations were reviewed by the study team and Patient and Public Involvement group before focus groups took part.	Methods - 5
18. Repeat interviews Were repeat interviews carried out? If yes, how many?	No	N/A
19. Audio/visual recording Did the research use audio or visual recording to collect the data?	Focus groups were recorded Microsoft Teams.	Methods - 5
20. Field notes Were field notes made during and/or after the interview or focus group?	No additional field notes were made.	N/A
21. Duration What was the duration of the interviews or focus group?	The focus groups lasted an average of 60 minutes.	N/A
22. Data saturation Was data saturation discussed?	We used information power	Methods - 5
23. Transcripts returned Were transcripts returned to participants for comment and/or correction?	No	N/A
**Domain 3: analysis and findings**		
**Data analysis**		
24. Number of data coders How many data coders coded the data?	One - LG	Methods - 5
25. Description of the coding tree Did authors provide a description of the coding tree?	N/A – not used	N/A
26. Derivation of themes Were themes identified in advance or derived from the data?	Themes were derived from the data	Methods - 5
27. Software What software, if applicable, was used to manage the data?	N/A	N/A
28. Participant checking Did participants provide feedback on the findings?	No	N/A
**Reporting**		
29. Quotations presented Were participant quotations presented to illustrate the themes / findings? Was each quotation identified? e.g. participant number	Yes	Results – 7-11

For peer review only

30. Data and findings consistent<br>Was there consistency between the data presented and the findings?	Yes	N/A
31. Clarity of major themes<br>Were major themes clearly presented in the findings?	Yes	Results 7-11
32. Clarity of minor themes<br>Is there a description of diverse cases or discussion of minor themes?	Yes	Results 7-11

For peer review only
